# Inflammatory Fibroblast‐Like Synoviocyte‐Derived Exosomes Aggravate Osteoarthritis via Enhancing Macrophage Glycolysis

**DOI:** 10.1002/advs.202307338

**Published:** 2024-02-11

**Authors:** Bin Liu, Yansi Xian, Xiang Chen, Yong Shi, Jian Dong, Lin Yang, Xueying An, Tao Shen, Wenshu Wu, Yuze Ma, Yi He, Wang Gong, Rui Peng, Jiaquan Lin, Na Liu, Baosheng Guo, Qing Jiang

**Affiliations:** ^1^ Division of Sports Medicine and Adult Reconstructive Surgery Department of Orthopedic Surgery Nanjing Drum Tower Hospital Affiliated Hospital of Medical School Nanjing University 321 Zhongshan Road Nanjing Jiangsu 210008 P. R. China; ^2^ State Key Laboratory of Pharmaceutical Biotechnology Nanjing University 22 Hankou Road Nanjing Jiangsu 210093 P. R. China; ^3^ Branch of National Clinical Research Center for Orthopedics Sports Medicine and Rehabilitation 321 Zhongshan Road Nanjing Jiangsu 210008 P. R. China; ^4^ Medical School of Nanjing University 22 Hankou Road, Gulou District NanJing Jiangsu 210093 P. R. China

**Keywords:** exosomes, fibroblast‐like synoviocytes, glycolysis, HIF1A, macrophages, osteoarthritis, synovitis

## Abstract

The severity of osteoarthritis (OA) and cartilage degeneration is highly associated with synovial inflammation. Although recent investigations have revealed a dysregulated crosstalk between fibroblast‐like synoviocytes (FLSs) and macrophages in the pathogenesis of synovitis, limited knowledge is available regarding the involvement of exosomes. Here, increased exosome secretion is observed in FLSs from OA patients. Notably, internalization of inflammatory FLS‐derived exosomes (inf‐exo) can enhance the M1 polarization of macrophages, which further induces an OA‐like phenotype in co‐cultured chondrocytes. Intra‐articular injection of inf‐exo induces synovitis and exacerbates OA progression in murine models. In addition, it is demonstrated that inf‐exo stimulation triggers the activation of glycolysis. Inhibition of glycolysis using 2‐DG successfully attenuates excessive M1 polarization triggered by inf‐exo. Mechanistically, HIF1A is identified as the determinant transcription factor, inhibition of which, both pharmacologically or genetically, relieves macrophage inflammation triggered by inf‐exo‐induced hyperglycolysis. Furthermore, in vivo administration of an HIF1A inhibitor alleviates experimental OA. The results provide novel insights into the involvement of FLS‐derived exosomes in OA pathogenesis, suggesting that inf‐exo‐induced macrophage dysfunction represents an attractive target for OA therapy.

## Graphical abstract

Exosome secretion from fibroblast‐like synoviocytes (FLSs) is elevated in synovium during osteoarthritis (OA). The exosomes from inflammatory FLSs lead to inflammation activation and M1 polarization in macrophages through HIF1A‐mediated hyperglycolysis, which further exert detrimental effects on chondrocytes and exacerbate OA. Inhibition of HIF1A can alleviate such macrophage dysfunction triggered by exosomes and attenuate experimental OA.

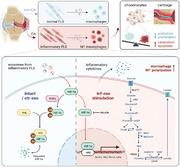

## 1 Introduction

As a leading cause of adult disability, osteoarthritis (OA) is the most prevalent joint disease, affecting over 240 million people worldwide and contributing to a 20% higher age‐adjusted mortality rate.^[^ ^1^ ^]^ Approximately 30% of individuals aged 45 years and older exhibit radiographic evidence of knee OA, with nearly half of them experiencing painful symptoms.^[^ ^2^ ^]^ Despite the substantial socioeconomic burden of OA, available therapies are limited to topical or oral nonsteroidal anti‐inflammatory drugs for pain alleviation. Joint arthroplasty surgery seems inevitable for patients with advanced‐stage OA.^[^ ^3^ ^]^ A poor understanding of OA pathogenesis hinders the development of efficacious therapies, thus necessitating immediate investigation.

Traditionally viewed as a wear‐and‐tear degeneration of the articular cartilage, it is now recognized as a whole‐joint disorder that affects various anatomical structures in and around the joint capsule, featured by subchondral bone sclerosis and synovial inflammation.^[^ ^4^ ^]^ Synovitis manifests as hypertrophy of the synovium, accompanied by an increased abundance of vascularity, villi, and fibrin deposits.^[^ ^5^ ^]^ MRI and ultrasonography studies suggest that synovial inflammation is not restricted to advanced‐stage OA and may even exceed cartilage damage in early OA.^[^ ^6^ ^]^ Baseline synovitis has prognostic value for accelerated structural deterioration of the cartilage.^[^ ^7^ ^]^ In addition, synovitis contributes to pain and swelling due to increased nerve infiltration and synovitis‐related effusion, two typical symptoms of OA.^[^ ^7^, ^8^ ^]^ Taken together, synovial inflammation is involved in both the onset and progression of OA, thus understanding its exact molecular mechanism, which remains elusive, may offer new opportunities for OA treatment.

To elucidate the synovial pathology in OA, attempts have been made to elaborate on the dynamics of synovial cellular composition. Fibroblast‐like synoviocytes (FLSs) are the most abundant cell type in both healthy and OA synovium, which primarily secrete lubricating factors to reduce friction and nourish cartilage chondrocytes.^[^ ^9^ ^]^ Following disturbances such as joint injury, Prg4^+^ synovial progenitor cells in the lining layer and cells adjacent to blood vessels in the sublining layer undergo significant expansion to replenish specialized FLSs, which macroscopically manifests as synovial lining hypertrophy.^[^ ^9^ ^]^ The expanded FLS population amplifies synovitis by facilitating immune cells infiltration and modulating angiogenesis and nerve growth.^[^ ^10^ ^]^ Targeting abnormal FLSs has demonstrated promising potential for OA treatment by reducing the detrimental pro‐catabolic and pro‐inflammatory paracrine signals that contribute to cartilage degeneration.^[^ ^11^ ^]^ However, the precise mechanism through which FLSs interacts with other joint cells remains unclear.

Another histological characteristic of synovitis is the increased infiltration of macrophages in the synovium.^[^ ^12^ ^]^ Macrophages, being the predominant leukocyte population in OA joint fluid and the most abundant immune cells in the synovium, constitute ≈ 40% of the synovial immune cell population in OA.^[^ ^13^ ^]^ Macrophages can display either a pro‐inflammatory M1 subtype or an anti‐inflammatory M2 subtype in response to various environmental stimuli, with the balance between them orchestrating the inflammatory and resolution phases following tissue injury.^[^ ^14^ ^]^ The activation state and ratio of M1/M2 macrophages are also strongly correlated with OA severity.^[^ ^15^ ^]^ Our previous single‐cell RNA sequencing analysis further validated the prevalence of M1 polarization over M2 polarization in the synovium of OA patients, with a dramatically expanded macrophage population.^[^ ^16^ ^]^ M1 macrophages interact with chondrocytes and aggravate cartilage destruction through the secretion of inflammatory cytokines, degenerative enzymes, and other factors, such as R‐spondin‐2.^[^ ^17^ ^]^ Therefore, targeting the excessive M1 polarization or reprogramming M1 macrophages into the M2 subtype emerges as a potential OA therapy.

Furthermore, immunometabolism is a burgeoning category that focuses on metabolic pathways and their effect on cell fate and immune function, which is also referred to as metabolic reprogramming.^[^ ^18^ ^]^ Macrophages exhibit distinct metabolic characteristics that correspond to their functional state.^[^ ^19^ ^]^ Generally, M1 macrophages are characterized by high levels of inducible nitric oxide synthase (iNOS) and display an enhanced glycolytic metabolism. In contrast, M2 macrophages primarily rely on the tricarboxylic acid cycle and oxidative phosphorylation (OXPHOS).^[^ ^20^ ^]^ Activated immune cells, including macrophages, demonstrate increased rates of glycolysis, resulting in increased glucose consumption and lactate production, similar to the Warburg effect in the tumor microenvironment.^[^ ^19^, ^21^ ^]^ This shift from OXPHOS to glycolysis leads to the production of metabolic intermediates and reactive oxygen species, which in turn promotes the synthesis of inflammatory mediators and matrix degenerative enzymes.^[^ ^22^ ^]^ However, limited research has revealed the mechanisms by which microenvironmental cues initiate immunometabolic reprogramming and promote the preferential polarization of synovial macrophages in OA.

Recently, exosomes, which are extracellular vesicles ranging from 40 to 160 nm in diameter, have gained attention as a new medium for intercellular communication. Exosomes plays an indispensable role in regulating proliferation, apoptosis, and inflammatory responses in multiple cell types during OA.^[^ ^23^ ^]^ Regarding macrophages, exosomes derived from osteoarthritic chondrocytes could promote inflammasome activation and augment mature interleukin 1 beta (IL‐1β) production in macrophages.^[^ ^24^ ^]^ In contrast, exosomes derived from normal chondrocytes or embryonic MSCs induce polarization towards an M2 phenotype and facilitate cartilage repair.^[^ ^25^ ^]^ However, there is a lack of research on the specific impact of FLS‐derived exosomes on macrophages in the context of OA.

In this study, we propose that inflammation spreads from FLSs to macrophages via exosomes, which promotes macrophage M1 polarization and exacerbates cartilage destruction. We first observed through clinical specimens and animal models that exosome secretion was upregulated in FLSs during OA, as well as IL‐1β‐stimulated FLSs in vitro. Exosomes derived from inflammatory FLSs induced classical activation of macrophages, resulting in the subsequent suppression of chondrogenesis in both a co‐culture system and an intra‐articular injection trial. Hyperglycolysis was subsequently validated as the underlying immunometabolic mechanism that triggered M1 polarization post exosome stimulation. Furthermore, HIF1A was identified as the master transcription factor governing exosome‐triggered glycolysis, and targeting HIF1A has shown potential for alleviating OA, partially by diminishing the spread of inflammation by exosomes.

## 2 Results

### 2.1 Exosome Secretion is Elevated in OA Synovium and Inflammatory FLSs

**Figure 1:**
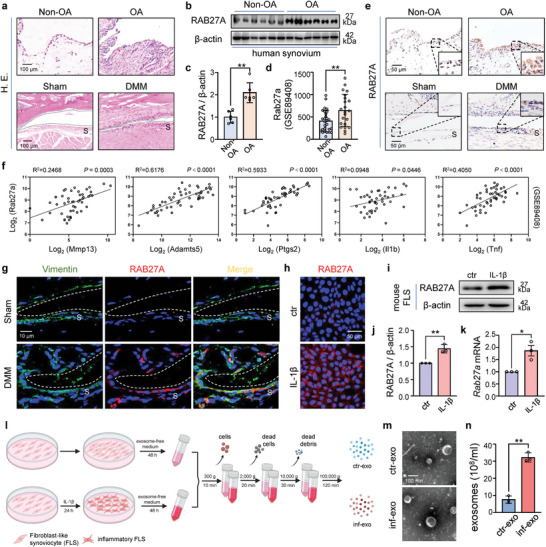
Exosome secretion from fibroblast‐like synoviocytes (FLSs) is elevated under inflammation stimulation. a) Representative images of HE staining of synovial specimens (synovium marked as S on the images) from patients with and without OA (top), and mice subjected to sham or DMM operation (bottom) after 8 weeks. Scale bar, 100 µm. b) Western blot analysis of RAB27A in synovial tissues from patients with or without OA, using β‐actin as loading control. c) Quantification of (b) using ImageJ. n = 6 per group. d) Gene expression of Rab27a reanalyzed using publicly available RNA‐seq results of GSE89408 from the Gene Expression Omnibus (GEO) database, including healthy (n = 28) and OA (n = 22) synovium biopsies. e) Representative images of immunohistochemistry staining of RAB27A of synovial specimens from patients with and without OA (top), and mice subjected to sham or DMM operation (bottom) after 8 weeks. Boxed area is enlarged in the picture corner. Scale bar, 50 µm. f) Pearson's correlation analysis of Rab27a expression with OA‐related matrix degradation enzymes (Mmp13 and Adamts5) and inflammatory genes (Il1b, Tnf, Ptgs2) based on the RNA‐seq results of GSE89408 from the GEO database. g) Representative images of immunofluorescence co‐staining of FLS marker vimentin (green) and RAB27A (red) for synovium 8 weeks after sham or DMM operation. Scale bar, 10 µm. h) Immunofluorescence staining of RAB27A in mouse FLSs stimulated by interleukin 1‐beta (IL‐1β) for 48 h. Scale bar, 50 µm. i) and j) Western blot analysis and quantification of RAB27A protein in mouse FLSs stimulated by IL‐1β for 48 h. k) RT‐qPCR analysis of messenger RNA levels for *Rab27a* in mouse FLSs after being stimulated by IL‐1β for 24 h. l) Experiment design diagram of exosome isolation procedures by sequential centrifugation from cell culture supernatant of FLSs stimulated with or without IL‐1β, referred to as inf‐exo or ctr‐exo respectively later. m) Representative images of exosomes by transmission electron microscopy. Scale, 100 nm. n) Quantification of the exosomes derived from control or inflammatory FLS culture supernatant by nanoparticle tracking analysis (NTA). Data presented as mean ± SD. ** *P* < 0.01, * *P* < 0.05, versus the indicated groups, Student's *t*‐test.

To confirm the presence of synovitis in OA, we analyzed the synovial specimens obtained from both human and mouse knees. Consistent with previous findings,^[^ ^17c^ ^]^ we observed marked synovial hyperplasia characterized by abundant cell infiltration in the OA synovium (**Figure** **1**a; Figure S1, Supporting Information). To investigate the role of synovial exosomes in OA, we analyzed the expression of RAB27A, a crucial regulator of exosome biogenesis that facilitates the docking of multivesicular bodies (MVBs) to the plasma membrane.^[^ ^26^ ^]^ We also found that RAB27A protein expression significantly increased in human OA synovium (Figure 1b,c). Additionally, the transcription level of Rab27a was elevated in the synovial RNA‐seq dataset (Figure 1d). RAB27A‐positive cells accumulated in both human OA synovium and mouse destabilization of the medial meniscus (DMM) synovium (Figure 1e). Furthermore, the expression of Rab27a was positively correlated with that of OA‐related markers (Figure 1f). These results indicate that the secretion of synovial exosomes is elevated in OA synovitis.

Considering the cellular composition of the OA synovium, FLSs are the predominant resident subpopulation, both in cell number and proportion.^[^ ^10^ ^]^ However, alternations in exosome secretion from FLSs during OA remains unclear. We observed an increased accumulation of RAB27A‐expressing FLSs in the mouse DMM synovium compared with that in the sham group (Figure 1g; Figure S2a, Supporting Information). To mimic the inflammatory microenvironment in OA joints, FLSs were stimulated with IL‐1β and showed elevated expression of genes encoding OA‐related enzymes, *Mmp3* and *Mmp13* (Figure S3, Supporting Information). We further found the protein and mRNA levels of RAB27A were elevated in mouse FLSs after IL‐1β stimulation (Figure 1h–k; Figure S2b, Supporting Information).

Furthermore, exosomes were isolated by sequential ultracentrifugation from the cell culture supernatant of FLSs treated with or without IL‐1β (Figure 1l). The morphologies of both exosomes were typical bilayer membrane‐bounded structures, with mean particle diameter of 139.2 nm for exosomes derived from intact FLSs (ctr‐exo) and 127.1 nm for exosomes derived from inflammatory FLSs (inf‐exo) (Figure 1m; Figure S4a, Supporting Information). Both the exosome groups contained common exosome markers (Figure S4b, Supporting Information). Interestingly, nanoparticle tracking analysis revealed that FLSs stimulated with IL‐1β secreted significantly more exosomes than intact FLSs (Figure 1n). Taken together, FLS exosome secretion was elevated under inflammatory stimulation both in vivo and in vitro.

### 2.2 Pathological Role of Inflammatory FLS‐Derived Exosomes in M1 Polarization

**Figure 2:**
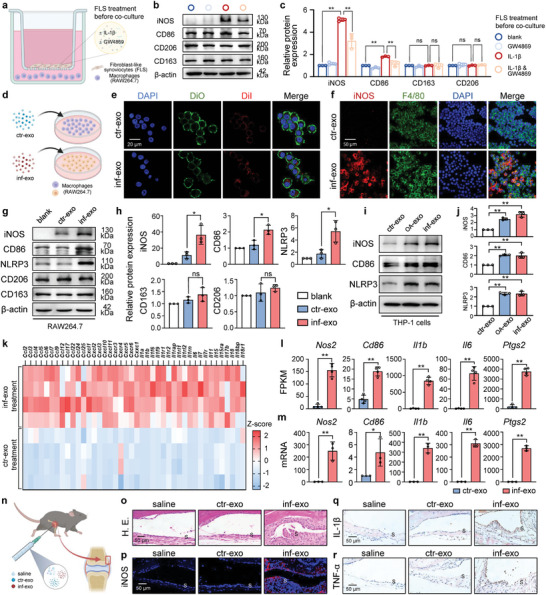
Exosomes from inflammatory FLSs promote macrophage classical activation in vitro and in vivo. a) Experiment design diagram of co‐culturing FLSs with RAW264.7 macrophages free of direct contact using Transwell apparatus. FLSs were treated with or without IL‐1β (10 ng mL^−1^) and GW4869 (20 µM) before co‐culture. b) and c) Western blot analysis and quantification of protein expression of iNOS, CD86, CD163, and CD206 in macrophages after co‐culturing with FLSs as indicated in (a). d) Schematic diagram for stimulating macrophages with ctr‐exo and inf‐exo in e) to f). e) Representative immunofluorescence images showing the uptake of DiI‐labelled exosomes (red) by DiO‐labelled macrophages (green) after incubation for 2 h. Scale bar, 20 µm. (f) Representative immunofluorescence staining of macrophage marker F4/80 (green) and M1 polarization marker iNOS (red) in macrophages stimulated by ctr‐exo and inf‐exo separately for 48 h. Scale bar, 50 µm. g) and h) Western blot analysis and quantification of protein expression of iNOS, CD86, NLRP3, CD163, and CD206 in RAW264.7 macrophages stimulated by ctr‐exo and inf‐exo separately. i) and j) Western blot analysis and quantification of protein expression of iNOS, CD86, and NLRP3 in THP‐1 macrophages stimulated by ctr‐exo and inf‐exo separately. k) Heatmap of selected chemokines and interleukins from the RNA‐seq results normalized using Z‐score. l) Expression of macrophage classical activation markers (Nos2, Cd86, Il1b, Il6, Ptgs2) in macrophages stimulated by ctr‐exo and inf‐exo from RNA‐seq results. m) RT‐qPCR for messenger RNA of inflammatory genes (*Nos2, Cd86, Il1b, Il6, Ptgs2*) in macrophages stimulated by ctr‐exo and inf‐exo. n) Diagram for intra‐articular injection of exosomes, the red rectangle indicating the synovial region for analysis. o) to r) Representative synovium images of hematoxylin and eosin (H.E.) staining, immunofluorescence staining for iNOS, and immunohistochemistry staining for IL‐1β and TNF‐α receiving injection of 20 µg exosomes suspended in 10 µL saline twice per week for 4 weeks. Scale bar, 50 µm. Data presented as mean ± SD. ** *P* < 0.01, * *P* < 0.05, ns, not significant, versus the indicated groups, Student's *t*‐test.

Synovitis is characterized by an increased accumulation of macrophages with a preference for classical activation, commonly referred to as M1 polarization.^[^ ^17c^ ^]^ The differentially expressed genes between synovium with or without OA are related to inflammation and immune response according to the Gene Ontology enrichment analysis (Figure S5a, Supporting Information). Compared to non‐OA controls, a significant increase in iNOS‐positive cells (a marker for M1‐like macrophages) and NLRP3‐positive cells (a marker for inflammasome activation) was observed in both human OA synovium and mouse DMM synovium (Figure S5b,c, Supporting Information). Given the close spatial proximity between FLSs and macrophages within the synovium, we hypothesized that exosomes derived from inflammatory FLSs disrupt macrophage polarization. FLSs were first pretreated with GW4869 to inhibit exosome secretion and then stimulated with recombinant mouse IL‐1β protein. Subsequently, FLSs were co‐cultured with RAW264.7 macrophages for 48 h (**Figure** **2**a). Compared to co‐culturing with intact FLSs, co‐culturing with inflammatory FLSs resulted in a significant upregulation of M1‐like macrophage markers (CD86 and iNOS). However, the expression of M2‐like markers, including CD163 and CD206, remained relatively unchanged. Interestingly, the inhibition of exosomes using GW4869 resulted in a diminishment in the increased expression of M1‐like macrophage markers induced by inflammatory FLSs (Figure 2b,c), indicating that exosomes derived from inflammatory FLSs facilitate macrophage polarization towards the M1 phenotype.

To further investigate the hypothesis that inf‐exo promote macrophage M1‐like polarization compared to ctr‐exo, we first confirmed the internalization of exosomes by macrophages (Figure 2d,e). Remarkably, macrophages stimulated by inf‐exo exhibited a noteworthy increase in the expression of M1‐like macrophage markers, as well as the inflammasome marker, in comparison to macrophages stimulated with ctr‐exo. Meanwhile, the expression of M2‐like macrophage markers was similar in macrophages stimulated by inf‐exo and ctr‐exo (Figure 2f–h). In parallel, we validated the observations made in murine cells again using human primary FLSs (Figure S6a,b, Supporting Information). We confirmed that FLSs isolated from OA joints, as well as IL‐1β stimulated non‐OA FLSs, maintained OA patterns in vitro, and then isolated exosomes from cell culture supernatants, followed by exosome characterization (Figure S6c–h, Supporting Information). Compared to non‐OA FLS‐derived exosomes (ctr‐exo), exosomes derived from OA FLSs (OA‐exo) and IL‐1β stimulated on‐OA FLSs (inf‐exo) were capable of inducing M1 polarization and inflammatory activation of human macrophages (Figure 2i,j; Figure S6i, Supporting Information).

To further confirm the pro‐M1‐polarization effect of inf‐exo, RAW264.7 cells were subsequently analyzed using RNA sequencing. A total of 1663 genes were significantly upregulated, while 1461 genes showed down‐regulation in macrophages stimulated with inf‐exo, compared to macrophages stimulated with ctr‐exo (Figure S7, Supporting Information). A cluster of classical chemokines and interleukins were significantly upregulated upon inf‐exo stimulation (Figure 2k). Additionally, the M1 macrophage marker genes *Nos2* and *Cd86*, as well as the typical inflammatory genes *Il1b*, *Il6*, and *Ptgs2*, exhibited a significant increase in expression levels upon exposure to inf‐exo, as confirmed by the quantitative polymerase chain reaction (qPCR) (Figure 2l,m). In contrast, the expression levels of M2‐related genes (*Mrc1*, *Arg1*, *Pparg*, and *Fizz1*) were similar in the macrophages after inf‐exo and ctr‐exo stimulation (Figure S8, Supporting Information)

We then administered intra‐articular injections of ctr‐exo, inf‐exo, or saline in equal volumes (Figure 2n). Similarly, inf‐exo injection resulted in a significant increase in synovial hyperplasia and cellular infiltration compared to both the saline and ctr‐exo injection synovium (Figure 2o). Elevated expression of the M1‐like macrophage marker iNOS and increased levels of the pro‐inflammatory cytokines IL‐1β and TNF‐α were observed in the synovium receiving inf‐exo injection (Figure 2p–r). These collective findings suggest that exosomes derived from inflammatory FLSs can induce M1‐like macrophage polarization, both in vitro and in vivo.

### 2.3 Inf‐Exo‐Primed Macrophages Induce OA‐Like Phenotype in Co‐Cultured Chondrocytes

**Figure 3:**
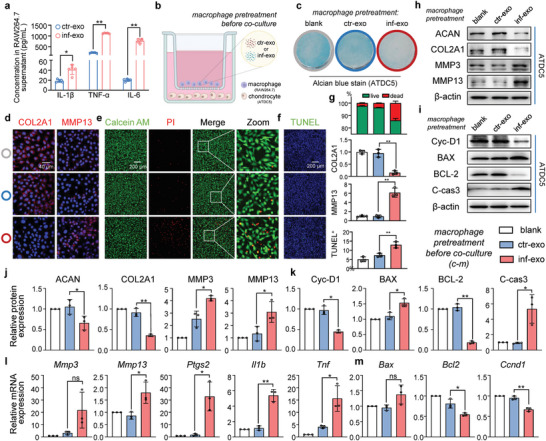
Inf‐exo‐primed macrophages promotes catabolism and apoptosis in chondrocytes. a) ELISA analysis of IL‐1β, IL‐6, and TNF‐α in macrophage culture supernatant after stimulated by inf‐exo or ctr‐exo. b) Experiment design diagram of co‐culturing mouse chondrocytes (ATDC5) with macrophages (RAW264.7) free of direct contact using the Transwell apparatus. Macrophages were stimulated by inf‐exo or ctr‐exo 24 h prior to co‐culture. c) Representative images of Alcian blue staining of chondrocytes after co‐culturing with exosome‐primed macrophages. The colored circles around the wells indicate the group for d) to f). d) Representative immunofluorescence images of COL2A1 and MMP13 in chondrocytes co‐cultured with exosome‐primed macrophages. Scale bar, 40 µm. e) and (f) Representative images of Live‐dead staining and TUNEL staining of chondrocytes after co‐culturing with exosome‐primed macrophages. Scale bar, 200 µm. g) Quantification of (d) to (f) using ImageJ software for three independent experiments. h) to k) Western blot analysis and quantification of ACAN, COL2A1, MMP3, MMP13, Cyclin D1, BAX, BCL2, and cleaved caspase‐3 in chondrocytes after co‐culturing with exosome‐primed macrophages. l) and m) RT‐qPCR for messenger RNA of *Mmp3, Mmp13, Il1b, Tnf, Ptgs2, Bax, Bcl2*, and *Ccnd1* in chondrocytes after co‐culture. Data presented as mean ± SD. ** *P* < 0.01, * *P* < 0.05, ns, not significant, versus the indicated groups, Student's *t*‐test.

Cartilage degeneration is the primary pathological manifestation of OA and is characterized by overactivated catabolism and apoptosis.^[^ ^27^ ^]^ Given the pathological role of macrophages in OA, we hypothesized that macrophages primed with inf‐exo may disrupt the behavior of co‐cultured chondrocytes. Increased levels of IL‐1β, IL‐6, and TNF‐α were detected in the cell culture supernatant of macrophages stimulated by inf‐exo, in comparison to macrophages stimulated with ctr‐exo (**Figure** **3**a). After co‐culturing using the Transwell apparatus, Alcian blue staining revealed a significant decrease in proteoglycan content in chondrocytes co‐cultured with inf‐exo primed macrophages (Figure 3b,c). Consistently, macrophages primed with inf‐exo downregulated anabolic markers (ACAN and COL2A1) in chondrocytes, whereas catabolic factors (MMP3 and MMP13) were upregulated (Figure 3d,g,h,j). Furthermore, macrophages primed with inf‐exo promoted the expression of inflammatory genes, such as *Ptgs2*, *Il1b*, and *Tnf*, along with extracellular degenerative enzymes, including *Mmp3* and *Mmp13* in co‐cultured chondrocytes (Figure 3l). Additionally, M1‐like macrophages induced by inf‐exo exhibited procatabolic effects similar to those induced by lipopolysaccharide (LPS), a classic and widely accepted model for inducing M1 polarization (Figure S9, Supporting Information).

Osteoarthratic chondrocytes experience a gradual decline in cell viability, resulting in an increasing number of vacant cellular lacunae in the cartilage. Co‐cultured chondrocytes with inf‐exo–primed macrophages displayed a reduced ratio of live to dead cells, as evidenced by calcein and propidium iodide staining (Figure 3e,g). Terminal deoxynucleotidyl transferase‐mediated dUTP nick‐end labeling (TUNEL) assay also demonstrated increased apoptosis in chondrocytes co‐cultured with inf‐exo–primed macrophages (Figure 3f,g). This was characterized by upregulated pro‐apoptotic proteins BAX and cleaved caspase‐3, and downregulated anti‐apoptotic protein BCL‐2, along with a halt in the proliferation marker cyclin‐D1 at both the transcriptional and translational levels (Figure 3i,k,m). Taken together, these findings indicate that macrophages primed with inf‐exo can induce an OA‐like phenotype in co‐cultured chondrocytes.

### 2.4 Intra‐Articular Injection of Inf‐Exo Accelerates Murine Experimental Osteoarthritis

**Figure 4:**
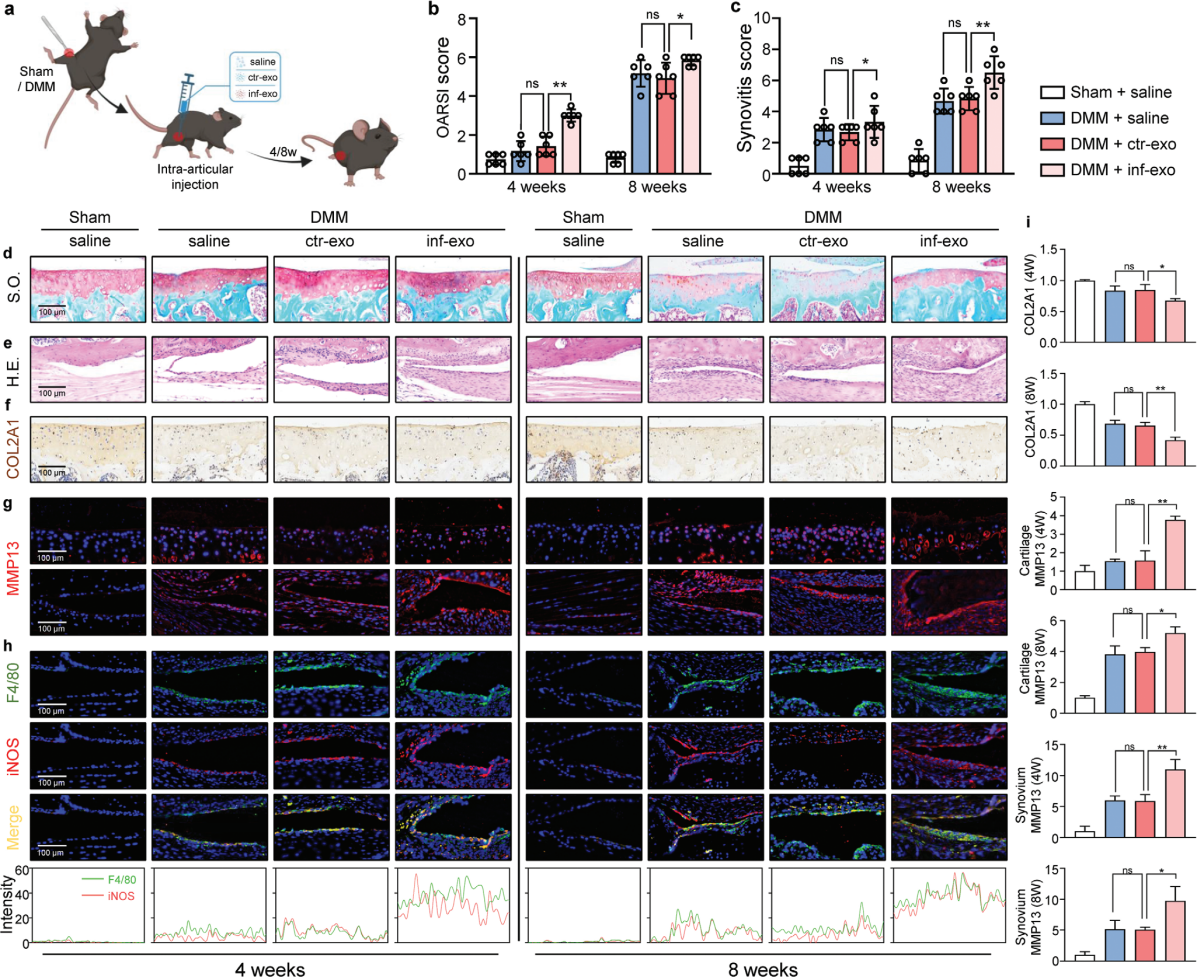
Intra‐articular injection of inf‐exo aggravates experimental osteoarthritis. a) Experiment diagram of intra‐articular injection of 20 µg inf‐exo or ctr‐exo resuspended in 10 µL PBS after DMM operation, twice a week for 4 and 8 weeks. b) and c) Quantitative analysis of Osteoarthritis Research Society International (OARSI) scale and synovitis score of mice described in (a). n = 6 per group. * *P*<0.05, student's *t*‐test. d) and e) Representative images of Safranin O‐fast green staining of joint cartilage area and H.E. staining of joint synovial membranes (n = 6). Scale bar, 100 µm. f) Representative images of immunohistochemistry staining of COL2A1 for joint cartilage (n = 6). Scale bar, 100 µm. g) Representative images of immunofluorescence staining of MMP13 in cartilage and synovium region (n = 6). Scale bar, 100 µm. h) Representative images and intensity quantification of immunofluorescence staining of F4/80 and iNOS in synovium region (n = 6). Scale bar, 100 µm. i) Quantification of intensity of IHC and IF staining in (f) and (g) using ImageJ. Data presented as mean ± SD. ** *P* < 0.01, * *P* < 0.05, ns, not significant, versus the indicated groups, Student's *t*‐test.

To investigate the in vivo role of inf‐exo or ctr‐exo in OA pathogenesis, specifically in cartilage degeneration and synovial inflammation, we established an experimental murine DMM model, followed by intra‐articular injection of exosomes for 4 or 8 weeks (**Figure** **4**a). Joints receiving inf‐exo injections exhibited deteriorated cartilage degradation, decreased COL2A1 staining, and heightened synovitis compared to joints receiving saline or ctr‐exo injections (Figure 4b–f,i). Increased matrix‐degrading enzyme MMP13 expression was noted in the cartilage and synovial membrane of the joints receiving inf‐exo injections (Figure 4g,i). Additionally, we observed synovial hyperplasia characterized by upregulated expression of the macrophage marker F4/80 and the M1 macrophage marker iNOS, as confirmed by double‐positive immunofluorescence staining (Figure 4h). Collectively, these findings suggest that exosomes derived from inflammatory FLSs play a significant role in expediting OA progression by promoting cartilage degradation and synovial inflammation.

### 2.5 Hyperglycolysis Triggered by Inf‐Exo Promotes M1‐Like Polarization and Macrophage Inflammation

**Figure 5:**
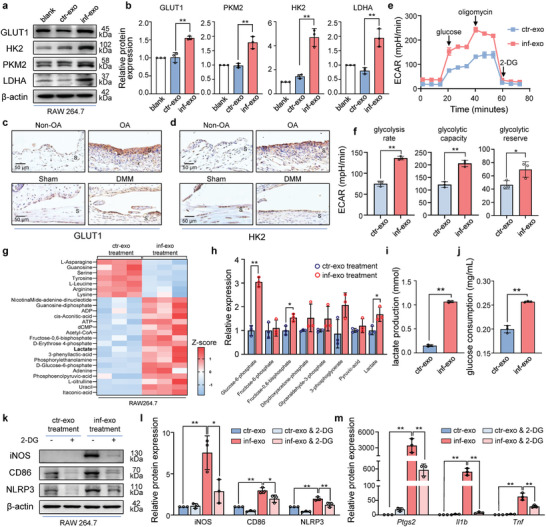
Inf‐exo‐triggered hyperglycolysis promotes macrophage inflammation. a) and b) Western blot analysis and quantification of GLUT1, HK2, PKM2, and LDHA in macrophages stimulated by ctr‐exo and inf‐exo, repeated in three independent experiments. c) and d) Representative images of immunohistochemistry staining for GLUT1 and HK2 in human (top panel) and mouse (bottom panel) synovium. Scale bar, 50 µm. e) and f) The extracellular acidification rate (ECAR) was measured using a XF96 Seahorse Analyzer, of which the glycolysis parameters were quantified. g) Heatmap of differentially expressed metabolites from the targeted metabolomics, fulfilling the criteria |log2 (fold change)| ≥ 1 and *P* < 0.05 (n = 3 each group). h) Relative concentration of glycolytic intermediates detected in targeted metabolomics using LC‐MS. Data shown in the histogram was normalized to those in macrophages stimulated by ctr‐exo. i) and j) Glucose consumption and lactate production in macrophage culture supernatants after exosome stimulation (n = 3 each group). k) and l) Western blot analysis and quantification of iNOS, CD86, and NLRP3 in macrophages stimulated by ctr‐exo and inf‐exo and glycolysis blockage using 2‐DG, repeated in three independent experiments. m) RT‐qPCR for messenger RNA of *Ptgs2, Il1b*, and *Tnf* in in macrophages stimulated by ctr‐exo and inf‐exo and blockage of glycolysis by 2‐DG (n = 3 each group). Data presented as mean ± SD. ** *P* < 0.01, * *P* < 0.05, versus the indicated groups, Student's *t*‐test.

After stimulating the macrophages with exosomes for 48 h, a significant alteration in the color of the cellular culture supernatant was observed (Figure S10a,b, Supporting Information), suggesting a variation in the pH levels of the supernatant resulting from exosome stimulation. Thus, we hypothesized that stimulation with inf‐exo, rather than with ctr‐exo, induces the activation of glycolysis in macrophages. Glucose transporter 1 (GLUT1), hexokinase 2 (HK2), pyruvate kinase M2 (PKM2), and lactate dehydrogenase A (LDHA) are the four key enzymes responsible for controlling glycolytic flux,^[^ ^19b^ ^]^ catalyzing four crucial reactions from glucose import to lactate efflux (Figure S10c, Supporting Information). Upon inf‐exo stimulation, the four aforementioned enzymes were significantly increased in macrophages compared to those in ctr‐exo‐treated macrophages, whereas these enzymes appeared similar in intact macrophages or macrophages stimulated by ctr‐exo (**Figure** **5**a,b). The elevation in glycolytic enzyme levels was consistent with the upregulated GLUT1 and HK2 in human OA synovium and mouse DMM synovium (Figure 5c,d). We further employed the Seahorse assay to investigate the status of cellular glycolysis, and observed significantly higher glycolytic rates and enhanced glycolytic capacity in macrophages treated with inf‐exo (Figure 5e,f).

We also conducted a targeted metabolomic analysis to investigate the metabolites associated with energy metabolism and found a general accumulation of glycolytic intermediates in macrophages stimulated with inf‐exo, among which glucose‐6‐phosphate and lactate exhibited a significant increase (Figure 5g,h). Similarly, elevated glucose consumption and lactate production were observed in the group subjected to inf‐exo stimulation (Figure 5i,j). Hence, glycolysis was activated in inf‐exo‐stimulated macrophages.

To investigate the necessity of glycolytic activation in macrophage M1‐like polarization and inflammatory activation induced by inf‐exo, we used 2‐DG to disrupt macrophage glycolysis prior to exosome stimulation. 2‐DG administration effectively suppressed the pro‐inflammatory effects of inf‐exo (Figure 5k,l). In parallel, the inhibition of glycolysis using 2‐DG resulted in the downregulation of inflammatory genes (*Ptgs2*, *Il1b*, *Tnf*) in macrophages stimulated with inf‐exo (Figure 5m). Taken together, these findings suggest that glycolysis may serve as the underlying mechanism responsible for M1‐like polarization and expression of inflammatory genes.

### 2.6 HIF1A is Identified as The Principal Transcription Factor Facilitating Hyperglycolysis and Polarization

**Figure 6:**
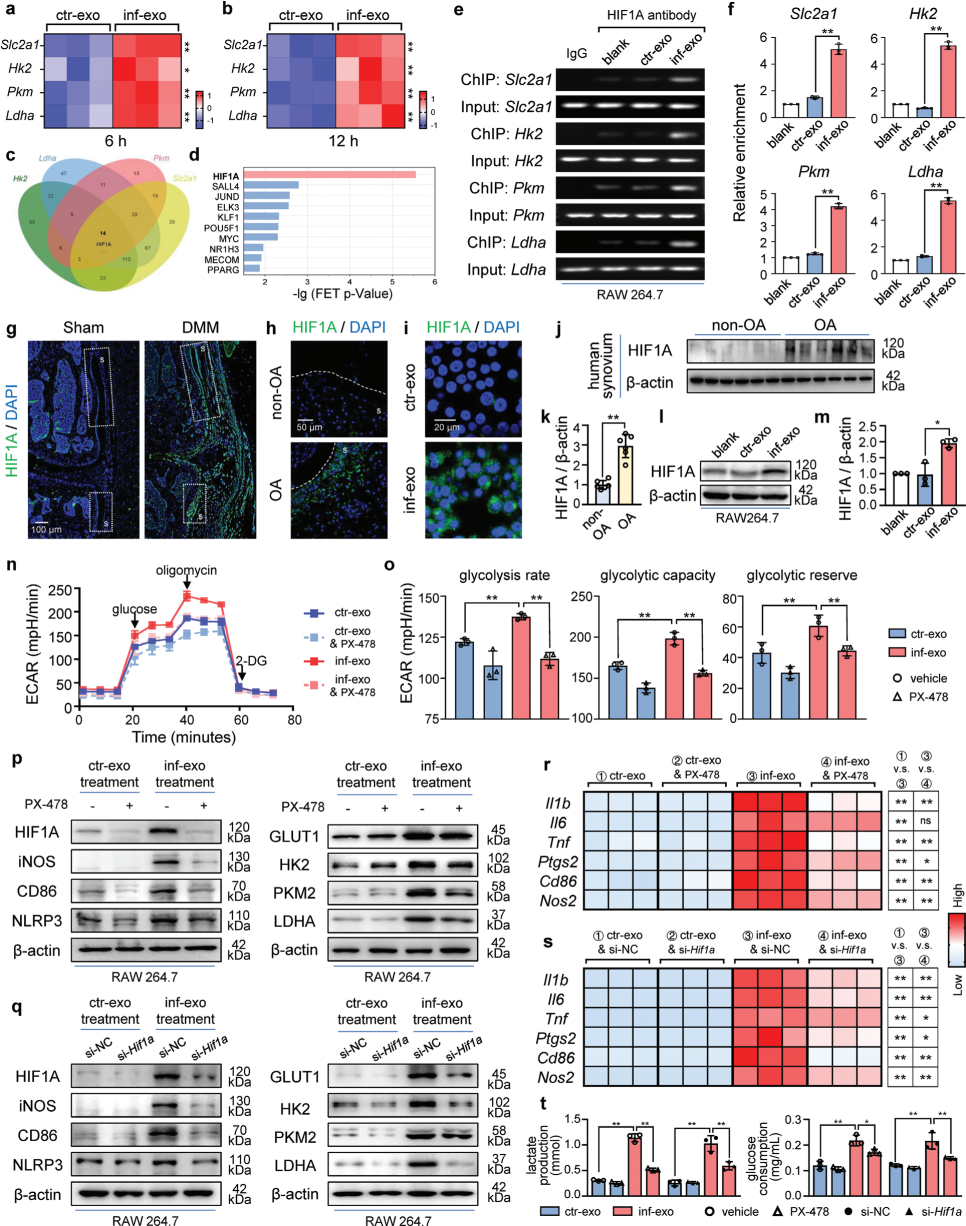
HIF1A is the key transcription factor which mediates inf‐exo‐triggered hyperglycolysis. a) and b) Heatmap of glycolytic gene messenger RNA levels (*Slc2a1, Hk2, Pkm*, and *Ldha*) in RAW264.7 stimulated by ctr‐exo and inf‐exo for 6 and 12 hours. c) Venn diagram of the transcription factor for four glycolytic genes predicted by ChIP base 3.0 database. d) Histogram diagram for predicted transcription factor ranking using ChEA3 database. e) and f) Representative agarose gel electrophoresis images and quantification for PCR products of ChIP assay of three independent experiments. The RAW264.7 cells as indicated were immunoprecipitated with the antibody against isotype‐matched immunoglobulin or HIF1A respectively 6 h after exosome stimulation. Then, the genomic DNA input and the antibody‐bound DNA fragments were amplified using PCR with primers covering the predicted sites on promoters of the four glycolytic enzymes. g) Representative HIF1A immunofluorescence staining of mice joint subjected to sham or DMM operation. The synovium region is boxed and labeled as S. Scale bar, 100 µm. h) Representative HIF1A immunofluorescence staining of clinical synovium specimens from patients with or without OA. Scale bar, 50 µm. i) Representative images of immunocytochemical staining for HIF1A of RAW264.7 cells stimulated by ctr‐exo and inf‐exo for 24 h. Scale bar, 20 µm. j) and k) Western blot analysis and quantification of HIF1A in synovial tissues from patients with or without OA, using β‐actin as loading control. n = 6 per group. l) and m) Western blot analysis and quantification of HIF1A in RAW264.7 macrophages stimulated by inf‐exo and ctr‐exo for 24 h. n) and o) ECAR was measured using a XF96 Seahorse Analyzer after exosome stimulation and HIF1A inhibition, of which the glycolysis parameters were quantified. p) and q) Western blot analysis of HIF1A, M1 polarization‐related markers (iNOS, CD86, and NLRP3), and glycolytic enzymes (GLUT1, HK2, PKM2, and LDHA) in macrophages stimulated by ctr‐exo and inf‐exo following genetical or pharmacological blockage of HIF1A. β‐actin was used as loading control. r) and s) Heatmap demonstration of macrophage inflammation‐related genes (*Il1b, Il6, Tnf, Ptgs2, Cd86*, and *Nos2*) in macrophages aforementioned. t) Glucose consumption and lactate production in cell culture supernatant after si‐RNA or PX‐478‐mediated blockage of HIF1A. Data presented as mean ± SD. ** *P* < 0.01, * *P* < 0.05, versus the indicated groups, Student's *t*‐test.

Consistent with the expression of glycolytic enzyme proteins, the four aforementioned glycolytic genes (*Slc2a1*, *Hk2*, *Pkm*, and *Ldha*) exhibited a significant increase in inf‐exo‐stimulated macrophages at 6 and 12 h (**Figure** **6**a,b). To elucidate the potential molecular mechanism underlying the activation of glycolytic gene transcription, we screened the upstream transcription factors for the four aforementioned glycolytic genes using the ChIP base 3.0 database and visualized them in a Venn diagram (Figure 6c). Additionally, HIF1A was identified as the top‐ranked transcription factor based on predictions from the ChEA3 database^[^ ^28^ ^]^ (Figure 6d). To verify the direct binding of HIF1A to the predicted site in the promoter region (Figure S11a, Supporting Information), namely the hypoxia response elements of the four glycolytic genes, a chromatin immunoprecipitation (ChIP) assay was conducted. Compared to macrophages stimulated by ctr‐exo, macrophages stimulated with inf‐exo exhibited a significant increase in the accumulation HIF1A in the promoter region (Figure 6e,f). Taken together, the findings of this study provide strong evidence supporting the notion that HIF1A plays a crucial regulatory role in the expression of glycolytic genes in macrophages stimulated with inf‐exo.

We then examined HIF1A expression under OA in vivo and in vitro. Elevated levels of HIF1A were observed in the synovial region of the mouse DMM synovium and human OA synovium compared to the non‐OA synovium (Figure 6g,h). Western blot analysis demonstrated upregulated HIF1A expression in the synovium of patients with OA (Figure 6j,k). Significant accumulation of HIF1A was observed in inf‐exo‐stimulated macrophages, whereas HIF1A was similar to non‐stimulated macrophages and those stimulated with ctr‐exo (Figure 6i,l,m).

To further illustrate the involvement of HIF1A in the activation of glycolysis and polarization of macrophages, we found that HIF1A inhibition using PX‐478 attenuated the activation of glycolysis induced by inf‐exo evidenced by Seahorse Assay (Figure 6n,o). The M1‐like polarization induced by inf‐exo and the upregulation of glycolytic enzymes were both attenuated by PX‐478 (Figure 6p; Figure S11e, Supporting Information). Additionally, HIF1A inhibition using PX‐478 effectively mitigated the inflammatory gene expression induced by inf‐exo (Figure 6r). Similar effects were also observed upon small interfering RNA (siRNA)‐mediated *Hif1a* knockdown (Figure 6q,s; Figure S11b–d,f, Supporting Information). Blockage of HIF1A also suppressed the aberrant elevation in glucose consumption and lactate production in inf‐exo‐stimulated macrophages (Figure 6t). Collectively, these findings demonstrate that the upregulation of HIF1A, triggered by inf‐exo stimulation, plays a crucial role in mediating macrophage hyperglycolysis and promoting M1‐like polarization.

### 2.7 Pharmacological Inhibition of HIF1A Attenuates OA In Vitro and In Vivo

**Figure 7:**
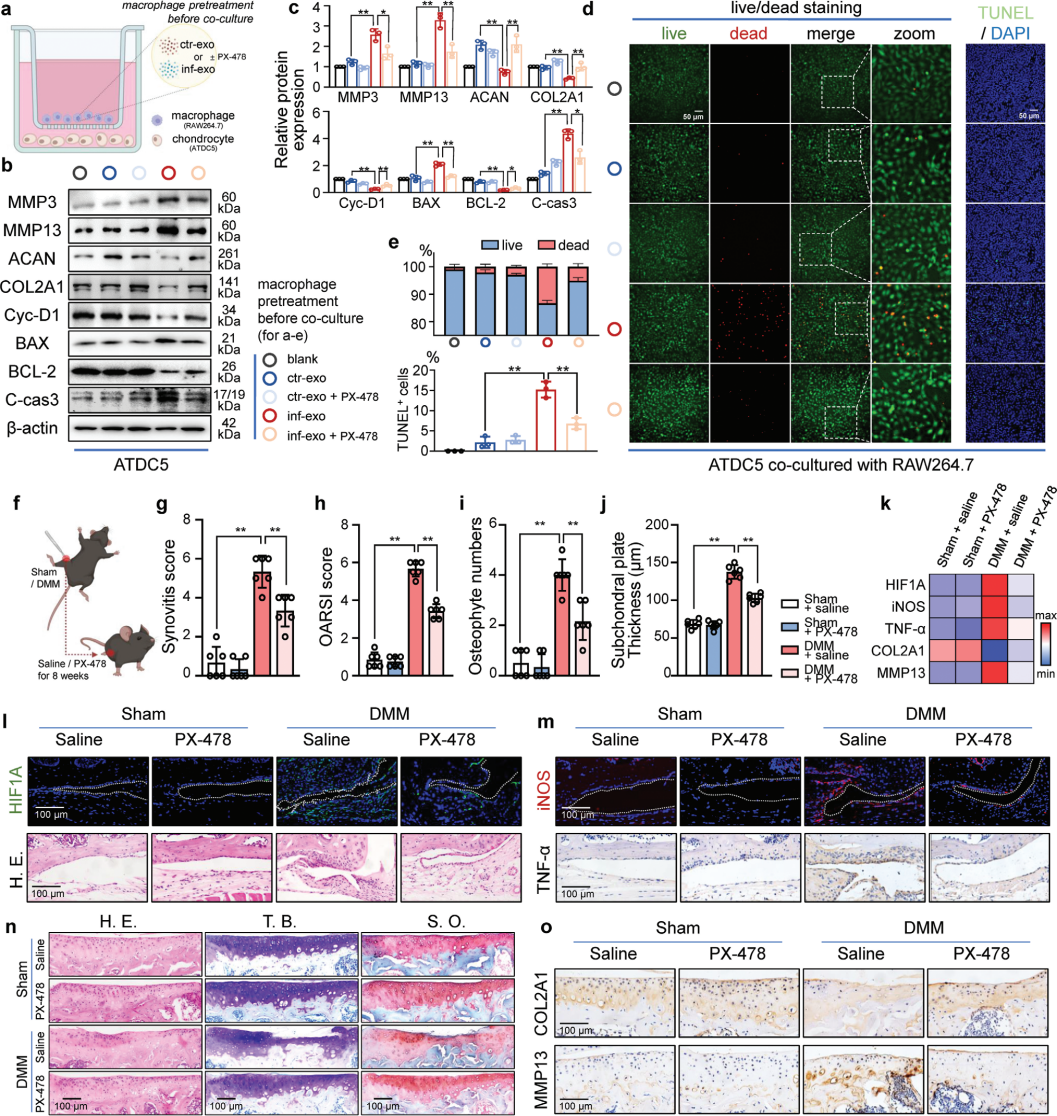
Pharmacological inhibition of HIF1A blunts inf‐exo‐triggered pathological role of macrophages. a) Scheme of the co‐culture apparatus for macrophages (RAW264.7) and chondrocytes (ATDC5) and the treatment for macrophages before co‐culture. Macrophages were stimulated by ctr‐exo or inf‐exo, with or without PX‐478 treatment. b) and c) Western blot analysis and quantification of OA‐related markers (MMP3, MMP13, ACAN, and COL2A1) and proliferation‐related markers (cyclin D1, BAX, BCL‐2, cleaved caspase‐3) in whole cell lysates of ATDC5 chondrocytes after co‐culture with RAW264.7 macrophages receiving corresponding pretreatment. Color circles indicate the groups assigned in (a) to (e). d) Representative images of live‐dead staining and TUNEL staining for ATDC5 cells after co‐culture with RAW264.7 cells. Scale bar, 50 µm. e) Quantitative analysis of live‐dead staining and TUNEL staining using ImageJ software for three independent experiments. f) Scheme for in vivo study of the effect of PX‐478, the selective HIF1A inhibitor, on murine OA. n = 6 each group. g) and h) Synovitis and OARSI score for joints at 8 weeks after sham or DMM operation. i) and j) Quantification of osteophyte numbers and subchondral bone thickness. k) Heatmap for the quantification results of the IF and IHC staining of synovium and cartilage, representing as Z‐score of the average intensity measured using ImageJ software (n = 6 each group). l) and m) Representative synovial images of H.E. staining, IF staining of HIF1A and iNOS, and IHC staining of TNF‐α. Scale bar, 100 µm. n) and o) Representative cartilage images of H.E., T.B., and S.O. staining, and IHC staining for COL2A1 and MMP13. Scale bar, 100 µm. Data presented as mean ± SD. ** *P* < 0.01, * *P* < 0.05, versus the indicated groups, Student's *t*‐test.

Our findings indicate that inf‐exo plays a crucial role in initiating macrophage M1 polarization through HIF1A‐mediated hyperglycolysis. Furthermore, inf‐exo‐primed macrophages induced an OA‐like phenotype in co‐cultured chondrocytes. To investigate the role of HIF1A in inf‐exo‐triggered macrophage dysfunction, HIF1A expression and activity in macrophages were inhibited using PX‐478 prior to inf‐exo stimulation and before co‐culturing with chondrocytes (**Figure** **7**a). The pro‐catabolic effect of macrophages on chondrocytes was significantly blunted when pretreated with PX‐478, as evidenced by a decrease in the production of matrix degradation enzymes (MMP3 and MMP13) and restoration of the expression of cartilage matrix components (COL2A1 and ACAN). Similarly, the expression of the cell proliferation marker cyclin‐D1 and the anti‐apoptotic protein BCL‐2 was restored in chondrocytes co‐cultured with inf‐exo‐primed macrophages pretreated with PX‐478, compared to those without PX‐478 treatment. Additionally, BAX and cleaved caspase‐3 levels were reduced in this coculture system (Figure 7b,c). Live/dead staining and TUNEL staining consistently demonstrated that PX‐478 pretreatment of macrophages could significantly diminish the adverse impact of inf‐exo on co‐cultured chondrocytes (Figure 7d,e), indicating that HIF1A could potentially serve as a promising target for OA therapy .

**Figure 8:**
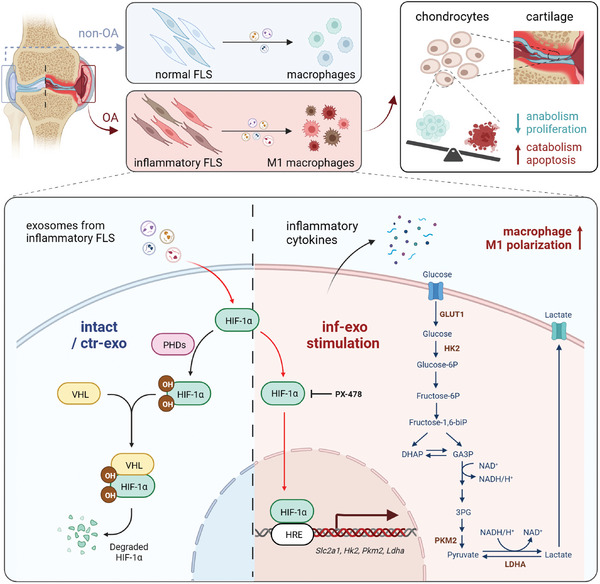
The scheme for exosomes derived from inflammatory fibroblast‐like synoviocytes which exacerbate osteoarthritis through promoting macrophage M1 polarization.

To further investigate the impact of inhibiting HIF1A on OA alleviation, we established a murine experimental OA model via the DMM procedure, followed by the administration of the HIF1A inhibitor PX‐478 (Figure 7f). Evaluation of serum liver and kidney function markers (ALT, AST, CREA, and BUN), along with histological examination of organ sections, indicated that the 8‐week administration of PX‐478 was safe in the murine model (Figure S12a,b, Supporting Information). Immunofluorescence staining of HIF1A revealed that PX‐478 effectively reversed the aberrant upregulation of HIF1A expression in the synovium region following the DMM procedure (Figure 7l,k). Synovial inflammation, as assessed by H.E. staining and synovitis scoring, was mitigated following PX‐478 administration (Figure 7g,l). iNOS and TNF‐α expression in synovial tissue of PX‐478‐treated DMM mice were also observed to be reduced (Figure 7m,k). Additionally, the administration of PX‐478 resulted in a reduction in cartilage degradation, evidenced by cartilage staining (Figure 7n), as indicated by a decrease in the OARSI score and restoration of COL2A1 expression, while the matrix proteinase MMP13 was suppressed (Figure 7h,k,o). We also observed that the administration of PX‐478 effectively mitigated the aberrant development of osteophytes and the thickening of the subchondral bone plate (Figure 7i,j; Figure S12c,d, Supporting Information). These findings suggest that injection of PX‐478 attenuates the development of experimental OA by inhibiting synovial HIF1A (**Figure** **8**).

## 3 Discussion

This study revealed a previously unidentified function of inflammatory FLS‐derived exosomes (inf‐exo) in OA‐related synovitis, particularly in facilitating macrophage M1 polarization. Essentially, inf‐exo spread inflammatory signals from FLSs to macrophages and aggravated synovitis, presenting novel targets for mitigating experimental murine OA. Briefly, inf‐exo stimulation leads to tightened glycolysis in macrophages, and inhibition of macrophage glycolysis alleviates inf‐exo–mediated M1 polarization. Mechanistically, inf‐exo stimulation leads to the accumulation of HIF1A, a transcription factor that modulates glycolysis, and mediates the upregulation of genes encoding glycolytic enzymes. Pharmacological inhibition or siRNA‐mediated genetic inhibition of HIF1A holds promise for controlling the spread of inflammation caused by inf‐exo, and injection of PX‐478 has been shown to mitigate OA in a murine model.

FLSs are the major cellular type in the synovial intima, and play a crucial role in coordinating intercellular communication within joints.^[^ ^10^ ^]^ In addition to their inflammatory and proliferating phenotypes following joint surface injury, distinct functional subsets of FLSs exert regulatory effects on various aspects of joint pathology, including cartilage degeneration, immune cell infiltration, angiogenesis, and neurogenesis.^[^ ^10^ ^]^ Consistent with previous research findings, we observed hyperplasia of the synovial lining layer in both synovium samples obtained from OA patients and DMM‐operated mice, accompanied by an upregulation in the expression of RAB27A, a crucial regulator of exosome secretion,^[^ ^26^ ^]^ in FLSs. Our in vitro study demonstrated that the inflammatory microenvironment induced by IL‐1β could also yield enhanced exosome secretion from FLSs.

Exosomes derived from IL‐1β stimulated FLSs have been found to induce osteoarthritic alterations in articular chondrocytes and facilitate tube formation in human umbilical vein endothelial cells.^[^ ^29^ ^]^ Similarly, FLS exosomes from OA patients can also lead to apoptosis of chondrocytes and degradation of the extracellular matrix, while exosomes from normal FLS could exert protective effects on chondrocytes.^[^ ^30^ ^]^ Exosomes derived from the synovial fluid of OA patients have the capacity to induce the release of pro‐inflammatory molecules by M1 macrophages in vitro.^[^ ^31^ ^]^ However, despite the close spatial proximity between FLSs and macrophages, the regulatory effect of inf‐exo on macrophages and the underlying mechanisms have not been precisely elucidated. During OA onset, the population of infiltrating macrophages undergoes a substantial increase in abundance and preferential polarization towards the pro‐inflammatory M1‐phenotype.^[^ ^10^ ^]^ Similarly, we observed M1‐like polarization in macrophages stimulated by inf‐exo, characterized by the upregulation of M1‐macrophage markers and inflammatory genes. The increased M1 polarization of macrophages leads to additional cartilage damage, partially through paracrine mechanisms. For instance, R‐spondin 2 secretion from activated M1 macrophage can activate the canonical Wnt/β‐catenin signaling pathway in chondrocytes, thereby exacerbating OA.^[^ ^17c^ ^]^ Similar to the pathogenic M1 macrophages in OA, the macrophages stimulated by inf‐exo in this study induced OA‐like patterns in co‐cultured chondrocytes, characterized by excessive catabolism and increased apoptosis. In murine experiments involving intra‐articular injection following DMM surgery, inf‐exo exacerbated synovitis characterized by elevated M1 macrophage infiltration and increased inflammatory cytokines, which may partially contribute to joint cartilage deterioration.

Regarding the mechanism underlying macrophage M1‐like polarization, we hypothesized from the perspective of immunometabolism, which highlights the dependence of immune cell phenotypes on the dynamics of metabolic pathways.^[^ ^18^ ^]^ Metabolic changes play a crucial role in determining the activation, differentiation, and functions of immune cells, with macrophages being no exception.^[^ ^18^ ^]^ Generally, M2 macrophages predominantly depend on OXPHOS, whereas M1 macrophages exhibit a shift toward glycolysis, even under normoxic conditions, resembling the Warburg effect in tumors.^[^ ^19^ ^]^ Despite being inferior to OXPHOS in terms of efficiency, glycolysis fulfills the heightened energy demands for biosynthesis in activated M1 macrophages, owing to its rapid ATP generation.^[^ ^32^ ^]^ Considering the significant role of glycolysis in inflammatory diseases, various strategies have been employed to modulate glycolysis and manipulate the pathogenic phenotype of macrophages, including synovitis during OA.^[^ ^33^ ^]^ In this study, we observed a significant increase in glycolysis following inf‐exo stimulation. Additionally, utilization of the glycolysis inhibitor 2‐DG mitigated inf‐exo‐induced M1 polarization and inflammatory activation. These findings suggest that enhanced glycolysis plays a role in M1 polarization induced by inf‐exo.

Several transcription factors are involved in the intricate and dynamic processes of immunometabolism. Our in‐silicon analysis identified HIF1A as the upstream regulator responsible for the upregulation of glycolytic enzymes, which was subsequently validated using a ChIP‐qPCR assay. In addition to its crucial involvement in cellular adaptation to hypoxic environments, targeting HIF1A holds attractive therapeutic potential for modulating the tumor microenvironment and immune cell function.^[^ ^34^ ^]^ Both hypoxia and LPS stimulation resulted in HIF1A accumulation in macrophages, which aligns with our findings in macrophages stimulated with inf‐exo.^[^ ^35^ ^]^ Depletion of HIF1A significantly dampens the aggregation, mobility, and bactericidal activity of macrophages and decreases ATP levels.^[^ ^36^ ^]^ This is because HIF1A is necessary for the adequate expression of several glycolytic genes, such as those encoding GLUT1, LDHA, HK2, and PKM2.^[^ ^34^ ^]^ In our in vitro experiments, the aberrant elevation of glycolytic enzymes and excessive M1 polarization were effectively suppressed by either pharmacological inhibition or genetic blockage of HIF1A along with reduced expression of inflammatory genes. This aligns with the findings of myocarditis research demonstrating that PX‐478 could alleviate the inflammatory responses by means of HIF1A inhibition.^[^ ^37^ ^]^ In addition, our study revealed that the administration of PX‐478, a commercially available inhibitor of HIF1A,^[^ ^38^ ^]^ exhibited therapeutic efficacy in a murine OA model, as evidenced by a decrease in the population of synovial iNOS‐expressing macrophages and ameliorated cartilage degeneration. Taken together, our investigation into the mechanism by which inf‐exo disrupts macrophage homeostasis presents a promising new target for experimental OA treatment.

The regulatory role of exosomes on HIF1A in recipient cells has recently attracted the attention of researchers. HIF1A can be selectively loaded into exosomes by LAMP2A, directly regulating the transcription of genes regarding angiogenesis and tumor invasion in target cells.^[^ ^39^ ^]^ Additionally, exosomes can also regulate the expression and activity of HIF1A through various non‐coding RNAs present in the exosomes, including microRNAs, circular RNAs, and long noncoding RNAs.^[^ ^40^ ^]^ However, considering the perplexity of exosomal cargoes, the exact mechanism underlying the accumulation of HIF1A in macrophages following inf‐exo stimulation and the key components within exosomes that trigger such effects remain unclear, warranting future research.

Additional limitations of the present study remain unresolved. First, the complexity of exosomes is reflected in the dynamic composition of exosome cargo, including proteins, lipids, and nucleic acids. Previous studies have demonstrated alterations in the cargo of exosomes derived from synovial fluid or FLS culture supernatants following inflammatory stimulation, including an increased concentration of pro‐inflammatory cytokines and altered non‐coding RNA profiles.^[^ ^29^, ^41^ ^]^ Further research is needed to clarify the pivotal exosomal cargo and bridge the gap between inf‐exo stimulation and HIF1A accumulation in macrophages. Once we identify the key components within inf‐exo, we could further perform in vivo gene‐editing experiments in FLSs and macrophages separately to further solidify such intercellular crosstalk mediated by aberrant exoosmes. Furthermore, this study primarily concentrated on the downstream impacts of inf‐exo. During exosome formation, we revealed that RAB27A, the protein regulating MVBs docking to the plasma membrane and formation of exosomes, was upregulated upon inflammation, and exosome release was elevated. However, the precise mechanism underlying heightened exosome secretion in response to inflammatory stimulation has yet to be investigated. As therapeutic interventions targeting abnormal exosome secretion have shown efficacy in various diseases, investigating the mechanisms underlying the excessive synthesis of exosomes by FLSs in OA holds great promise for clinical applications.^[^ ^42^ ^]^ Additionally, in the in vivo experiment section, we systematically administered the HIF1A inhibitor PX‐478. More than amplifying inflammation in macrophages, HIF1A also has a dismissible effect on chondrocyte homeostasis.^[^ ^43^ ^]^ Although no adverse effects on the cartilage were observed in the sham‐operated mice receiving PX‐478 injection, future experiments may necessitate the use of macrophage‐specific HIF1A knockout mice (such as *Lyz2*‐Cre mice) or the construction of a macrophage‐targeted smart delivery system for HIF1A inhibition for more precise intervention.

## 4 Conclusion

Our study demonstrates that exosomes derived from inflammatory FLSs play a crucial role in OA pathogenesis by facilitating M1 polarization of macrophages. From the perspective of intercellular crosstalk, exosomes spread inflammation from aberrant FLSs to macrophages, resulting in the activation of macrophages and amplification of synovitis. Consequently, inf‐exo‐primed M1‐like macrophages further render osteoarthritic phenotypes in chondrocytes, exacerbating OA. We also elaborate on the downstream mechanism of inf‐exo‐triggered macrophage dysfunction from an immunometabolic perspective. Inf‐exo stimulation enhances macrophage glycolysis via the HIF1A‐mediated transcription of glycolytic genes, satisfying the upregulated energy demands for inflammation activation. Further genetical or pharmacological blockage of HIF1A shows alleviation for inf‐exo–triggered macrophage aberrant polarization and attenuation for experimental murine OA. Taken together, our investigation emphasizes the involvement of inf‐exo in accelerating OA and indicates that inf‐exo‐triggered macrophage dysfunction may provide a novel avenue for OA therapy.

## 5 Experimental Section

### Animal Experiments

All male C57BL/6J mice were purchased (GemPharmatech Co., Ltd) and kept housed in the experimental animal center at the Medical School of Nanjing University. DMM operation was used as the murine experimental OA model as previously described,^[^ ^44^ ^]^ in which the medial meniscus was destabilized by transecting the medial meniscotibial ligament.

To assess the effect of ctr‐exo or inf‐exo on synovitis and cartilage degeneration, exosomes resuspended in saline were sterilized with a 0.22 µm filter and intra‐articularly injected into joint cavities (20 µg per mouse at each injection). To evaluate the therapeutic potential of HIF1A inhibition against OA, PX‐478 (B6004, APExBIO, Houston, USA) was administrated via intraperitoneal injection to sham or DMM mice PX‐478 (100 mg kg^−1^) every other day. Mice injected with an equal volume of valine were used as controls in each experiment.

Mice were euthanized by intraperitoneal injection of sodium pentobarbital at an overdose. Experimental protocols for animal experiments were approved by the Ethics Committee and the Institutional Animal Care and Use Committee of the Affiliated Nanjing Drum Tower Hospital, Nanjing University Medical School.

### Human Samples

Human samples were collected from patients at the Affiliated Nanjing Drum Tower Hospital of Nanjing University Medical School. Patients were generally divided into OA and non‐OA groups. The OA group was comprised of 6 patients who underwent total knee arthroplasty, while the non‐OA group included patients diagnosed without OA. The samples for historical analysis were fixed in 4% paraformaldehyde and other were stored at −80 °C before further protein analyses. For isolation of primary human FLSs, the synovium was processed immediately after operation. The study was approved by the Ethics Committee of Nanjing Drum Tower Hospital, and written informed consent was obtained from all patients. None of the patients was involved in the design, conduct, or interpretation of the study.

### Cell Culture

To obtain mouse FLSs, the synovial tissue of the knee joints of C57BL/6J mice was separated using microscopic scissors and forceps as previously described under a stereo zoom microscope.^[^ ^11b^ ^]^ After clearing the attached fat and other tissues, the obtained synovial tissue was subjected to enzymic digestion using 1 mg ml^−1^ Collagenase IV (17 104 019, Gibco, USA) and 0.1 mg ml^−1^ DNase I (AM2222, Invitrogen, USA) for 40 minutes at 37 °C. The digestion buffer was then passed through a 70 µm filter (abs7232, absin) and cells were collected using centrifugation at 300 ×g for 5 minutes. The cells were cultured in Dulbecco's Modified Eagle Medium (DMEM, 319‐005‐CL, WISENT) supplemented with 10% fetal bovine serum (FND500, ExCell Bio) and 2% 100x penicillin‐streptomycin solution (BC‐CE‐007, BioChannel). To mimic the inflammatory microenvironment during OA, FLSs were stimulated with 10 ng Ml^−1^ recombinant mouse IL‐1β protein (C042, Novoprotein, Shanghai) for inducing inflammation. For isolation of primary human FLSs, synovium collected from patients with or without OA was carefully separated and the following procedures were the same as those for mouse FLSs.^[^ ^11b^ ^]^

The Immortalized mouse cell line RAW264.7 (CL‐0190, Procell Life Science & Technology) and ATDC5 pre‐chondrocytes (ZQ0938, Zhong Qiao Xin Zhou Biotechnology) were purchased and maintained in medium described above. The cells at 90% confluence were detached using 0.25% trypsin‐EDTA solution (BC‐CE‐005, BioChannel) and passaged for experiments or frozen using a commercial cell‐saving media (C3520, VivaCell), and stored in liquid nitrogen. The immortalized human monocyte cell line THP‐1 cells (SNL‐044, Sunncell) were suspended in DMEM media and induced for macrophage differentiation using 10 ng ML^−1^ PMA (HY‐18739, MCE) for 24 hbefore exosome stimulation. And cells frozen using a rapid cell‐saving media (AC05L033, Life‐iLab, China) for maintenance.

For the Alcian Blue staining, the ATDC5 cells were additionally supplemented with 100 NM of dexamethasone (D4902, Merck), 1% Insulin‐Transferrin‐Selenium Media Supplement (C0341, Beyotime), 50 ng ml^−1^ of ascorbic acid (A4544, Merck), 10 ng ml^−1^ of TGF‐β1 (CK33, Novoprotein, Shanghai) for chondrogenesis differentiation. The cells were fixed using 4% paraformaldehyde at room temperature and then stained according to the manufactures’ instructions (KGA4102‐50, KeyGEN BIOTECH).

For blockage of *Hif1a*, small interfering RNA (siRNA) was transfected into RAW264.7 cells using FuGENE HD Transfection Reagent (Promega) before exosome stimulation, using sequence in Table S1, Supporting Information.

### In Vitro Co‐Culture Assays

Transwell Permeable Supports (3415, Corning, and TCS001012, Jet Biofil) were utilized to establish the co‐culture system. Briefly, 5 × 104 RAW264.7 cells were counted by Countstar (IC1000, Countstar) and seeded in a 24‐well plate 6 hour prior to co‐culture. Then an equivalent number of FLSs pretreated with or without IL‐1β and GW4869 (HY‐19363, MedChem Express) 24 hours prior to co‐culture were seeded in the top chamber of the insert apparatus, which was directly placed on the top of a well containing RAW264.7 cells, where the latter were harvested later for further analysis. Likewise, a similar co‐culture system was established with RAW264.7 cells on the top chamber and ATDC5 cells in the plate well was established.

### Exosome Isolation and Characterization

For exosome isolation, mouse FLSs were cultured as described above with or without IL‐1β for 24 h. The culture media were then replaced with high glucose DMEM supplemented with exosome‐free fetal bovine serum (C3801, VivaCell) to exclude unwanted exosome contamination, no longer adding recombinant mouse IL‐1β. Cell culture supernatants were collected for subsequent exosome extraction after 48 h. The exosomes from cell culture supernatant were collected by multi‐step centrifugation as described previously,^[^ ^45^ ^]^ using an L‐80 XP ultracentrifuge (Beckman Coulter).

The protein concentration of exosome samples was determined by the BCA assay (E‐BC‐K318‐M, Elabscience) and subjected to western blot to confirm the presence of exosomal markers. The number and size distribution of exosomes were tested by nanoparticle tracking analysis. The morphology of exosomes was examined using a transmission electron microscope.

### Western Blot Assay and Enzyme‐Linked Immunosorbent Assay (ELISA)

Proteins were extracted from the collected cells or ground synovium using pre‐cooled RIPA lysis buffer supplemented with a protease/phosphatase inhibitor cocktail and PMSF (KGP2100, KeyGEN BioTECH). Equal protein extracts were subjected to SDS‐PAGE, transferred to polyvinylidene fluoride membranes, blocked with 5% bovine serum albumin, and incubated with diluted primary antibody overnight.

All the primary antibodies were validated by either the manufacturer or other available publications, listed as follows: RAB27A (69 295), cleaved‐caspase3 (9661), and HIF1A (36 169) from Cell Signaling Technology. CD86 (A1199), iNOS (A3774), BAX (A0207), BCL‐2 (A0208), GLUT1 (69 295), and β‐actin (AC026) from ABclonal. CD206 (18704‐1‐AP), CD163 (16646‐1‐AP), Cyclin‐D1 (60186‐1‐Ig), HK2 (22029‐1‐AP), NLRP3 (27458‐1‐AP), MMP3 (66338‐1‐Ig), and MMP13 (18165‐1‐AP) from Proteintech. PKM2 (AF10640) and LDHA (AF14751) from AiFang Biological. COL2A1 (BA0533) from BOSTER. ACAN (NB600‐504) from NOVUS. After incubating with a horseradish peroxidase (HRP)‐conjugated secondary antibody (AS014 and AS003, ABclonal) and visualized using a commercial enhanced chemiluminescence kit (P2300, New Cell & Molecular Biotech), Grey values of the obtained bands were quantified using ImageJ software and normalized to expressions of β‐actin as loading control.

ELISA was utilized to quantify concentrations of pro‐inflammatory factors IL‐1β (SEKM‐0002, Beijing Solarbio Science & Technology Co., Ltd.), IL‐6, and TNF‐α (RK00008 and RK00027, ABclonal) in the cell culture supernatant, using commercial kits according to the manufacturer's instructions.

### Seahorse Glycolysis Stress Test

The Seahorse assay was performed to evaluate the capacity of glycolytic pathway according to the manufacturers’ instruction (103 020, Agilent Technologies). RAW264.7 macrophages (30000 cells/well) were seeded in Seahorse XF96 Cell Culture Microplates using DMEM medium (SP032010500, SPERIKON) adding 10% exosome‐free fetal bovine serum at 37°C incubator. The cells were stimulated with ctr‐exo, inf‐exo, with or without PX‐478 after adhesion. After 12 h, the medium was replaced with pre‐warmed XF‐basal medium supplemented with 2 mM L‐Glutamine followed by incubation in non‐CO2 incubator for 1 h before Seahorse assay. After measuring baseline extracellular acidification rate (ECAR), cells were sequentially treated with 10 mM glucose, 1 mM oligomycin, and 50 mM 2‐deoxyglucose (2‐DG). The data were analyzed using Wave Desktop 2.6 (Agilent Technologies).

### Quantitative Real‐Time Polymerase Chain Reaction (qRT‐PCR)

Total RNA was extracted using the FastPure Cell/Tissue Total RNA Isolation Kit V2 (RC112‐01, Vazyme Biotech Co.,Ltd) according to the standard protocol from manufacturer's protocol. The first‐strand cDNA was synthesized from the total RNA using the HiScript IV RT SuperMix for qPCR (R423‐01, Vazyme Biotech Co.,Ltd). Quantitative reverse transcription PCR was performed using the ChamQ SYBR qPCR Master Mix (Q311‐02, Vazyme Biotech Co.,Ltd) on a QuantStudio 5 Real‐Time PCR System (Thermo Fisher Scientific, USA). Relative expressions of the target genes were analyzed using the comparative C_T_ method with *Actb* as the internal standard. The primers were synthesized (Generay Biotech, Shanghai, China) using the sequences shown in Tables S3 and S4 (Supporting Information).

### Histological Analyses

Clinical synovium specimens and decalcified mouse knees were fixed in 4% paraformaldehyde, followed by dehydration before embedding in paraffin to make 5‐µm sections. Sections were deparaffinized with xylene, dehydrated with gradient ethanol and then stained with H.E. (hematoxylin and eosin, G1120, Solarbio), T.B. (Toluidine bule, JL‐R4922, Jonln), or S.O. (Safranin O‐Fast Green, G1371, Solarbio). The OARSI cartilage scoring system and synovitis scores were used as previously described.^[^ ^17^, ^46^ ^]^

### Immunohistochemistry (IHC) and Immunofluorescence Analyses (IF)

Deparaffinized and rehydrated sections were immersed in pepsin buffer for antigen retrieval. The sections for IHC staining were incubated with 3% hydrogen peroxide for 15 min. All sections were blocked with 1% goat serum containing 0.15% Triton X‐100 for 1 h followed by incubation with the primary antibodies diluted with a universal antibody diluent (WB100D, New Cell & Molecular Biotech) at 4°C overnight: vimentin (60330‐1‐Ig), TNF‐α (60291‐1‐Ig), IL‐1β (26048‐1‐AP), HK2 (22029‐1‐AP), HIF1A (20960‐1‐AP), and MMP13 (18165‐1‐AP) from Proteintech. GLUT1 (69 295) and iNOS (A3774) from ABclonal. COL2A1 (BA0533) from BOSTER. F4/80 (71 299) from Cell Signaling Technology. RAB27A (PA5‐79904) from Invitrogen.

For IHC staining, sections were incubated with HRP‐conjugated secondary antibodies and then stained with 3,3′‐diaminobenzidine. For IF staining, sections were incubated with fluorescent dye‐labeled secondary antibodies (SA00013‐1/4, Proteintech). Images of IHC slides were captured using an Olympus BX53 microscope, and images of IF slides were captured using a Leica DMi8 THUNDER Imaging Systems or Olympus FV3000 laser confocal microscope. The regions used for histological analysis were the medial tibial plateau in mice and the synovial surface areas in both human and murine specimens, with semi‐quantitative analysis conducted following methods as described in previously published literature.^[^ ^47^ ^]^

For immunocytochemical (ICC) staining, cells were seeded onto the glass bottom culture dishes (801 002, NEST Biotechnology) or chamber slides (1 092 000, SAINING) and subjected to the corresponding treatment.

### Micro‐X‐Ray Computed Tomography (µCT) Analysis

The micro architecture of the knees was analyzed using a µCT scanner (VivaCT80; Scanco Medical AG, Switzerland). After fixation using 4% PFA at room temperature overnight, the knees were scanned at 70 kV, 114 µA, and a resolution of 15.6 µm per pixel and the 3D reconstruction images were acquired with Scanco Medical software as previously described.^[^ ^48^ ^]^

### Chromatin Immunoprecipitation (ChIP) Assay

The RAW264.7 cells were fixed using formaldehyde (F809702, Macklin) after corresponding treatment. ChIP assay was performed using a Sonication ChIP Kit (RK20258, ABclonal). The prepared chromatin‐protein complexes were immunoprecipitated using protein A/G magnetic beads (HY‐K0202, MedChem Express) coated with anti‐HIF1A antibody (36 169) or anti‐IgG antibody (3900) purchased from Cell Signaling Technology. DNA was released from the chromatin‐protein complex and purified using FastPure Cell/Tissue DNA Isolation Mini Kit (DC102‐01, Vazyme). Diluted DNA was futher subjected to PCR using primers (listed in Table S2, Supporting Information) designed according to ChIP Base 3.0 database prediction,^[^ ^49^ ^]^ and quantified using agarose gel electrophoresis, followed by staining with GelRed (TSJ002, Tsingke Biological Technology). A Venn diagram for demonstrating the common transcription factor was generated using an online program (cloud.genepioneer.com).

### Live/Dead staining and TUNEL Staining

The Live & Dead Viability Assay Kit for Animal Cells (EFL‐CLD‐001, Engineering For Life) was utilized to distinguish live cells and dead cells using calcein AM and propidium iodide. Terminal deoxynucleotidyl transferase dUTP nick end labeling (TUNEL) staining was utilized to detect the DNA breaks during apoptosis using the One‐step TUNEL FITC Apoptosis Detection Kit (K1133, APExBIO, Houston, USA). The cells were imaged at the corresponding wavelengths.

### Biochemical Assays

Lactate production and glucose consumption were calculated by measuring the changes in lactate and glucose concentration in cell culture supernatant, using commercial kits following the manufacturer's instructions (AKAC001C and AKSU001C, Beijing Boxbio Science & Technology Co.,Ltd.).

Serum indicators of liver and kidney functions were tested to verify the safety of the PX‐478 injection, including alanine aminotransferase (ALT), aspartate aminotransferase (AST), creatinine (CREA), and blood urea nitrogen (BUN), using commercial kits according to manufactures’ instructions (C009‐2‐1, C010‐2‐1, C011‐2‐1, and C013‐2‐1, NJJCBIO).

### Analysis of Publicly Available Datasets and RNA Transcriptome Sequencing (RNA‐seq)

The mRNA expression of Rab27a and other OA‐related genes was obtained from the Gene Expression Omnibus (GEO) database (dataset GSE89408) and analysed using the online website eVITTA.^[^ ^50^ ^]^ For RNA‐seq, total RNA was isolated from RAW264.7 macrophages after ctr‐exo or inf‐exo separately. The paired‐end library was prepared and then sequenced on an Illumina Novaseq platform, conducted by Novogene Biotech (Beijing, China).

### Targeted Metabolomic Profiling

Macrophages stimulated with ctr‐exo or inf‐exo were collected and quick‐frozen in liquid nitrogen. Resuspened using methanol, the metabolities were extracted by freeze‐thaw cycle and analyzed using a liquid chromatography‐electrospray tandem mass spectrometry (LC‐ESI MS/MS) system (EClassical 3200, Waters ACQUITY H‐Class and QTRAP 6500+ System). The targeted metabolomics analysis was conducted by Allwegene (Beijing, China).

### Statistical Analysis

Data were demonstrated as means ± SDs and analyzed with GraphPad Prism software (version 9.4), of which the *P* < 0.05 was considered significant. The data were obtained from results of at least three independent experiments. A *t* test was utilized to analyze the differences between groups and a one‐way analysis of variance (ANOVA) test with Tukey's post hoc test was utilized for assessing the differences of mean values of more than two groups.

## Conflict of Interest

The authors declare no conflicts of interest.

## Author Contributions

B.L., Y.S.X., and X.C. contributed equally to this work. B.L. and Y.S.X. conducted majority of the assays, acquired, and analyzed data, and drafted the manuscript. J.D., Y.S., X.C., and X.Y.A. assisted in project design and provided technique supports. W.S.W., W.G., T.S., R.P., and N.L. participated in animal experiments. Y.Z.M., Y.H., and J.Q.L. participated in exosome‐related experiments. Q.J. and B.S.G. conceived, designed the project, supervised experiments, and provided research resources. All authors approved the final version of this manuscript.

## Supporting information

Supporting Information

## Data Availability

The data that support the findings of this study are available in the supplementary material of this article.
