# Supplementary material for: Inflammatory Fibroblast‐Like Synoviocyte‐Derived Exosomes Aggravate Osteoarthritis via Enhancing Macrophage Glycolysis
Source: Adv Sci (Weinh). 2024 Feb 11;11(14):2307338. doi: 10.1002/advs.202307338 (PMC11005727; doi:10.1002/advs.202307338)
Supplement: Supplementary file 1 — Supporting Information [file ADVS-11-2307338-s001.pdf]

ADVANCED SCIENCE
Open Access

Supporting Information

for *Adv. Sci.*, DOI 10.1002/advs.202307338

Inflammatory Fibroblast-Like Synoviocyte-Derived Exosomes Aggravate Osteoarthritis via Enhancing Macrophage Glycolysis

Bin Liu, Yansi Xian, Xiang Chen, Yong Shi, Jian Dong, Lin Yang, Xueying An, Tao Shen, Wenshu Wu, Yuze Ma, Yi He, Wang Gong, Rui Peng, Jiaquan Lin, Na Liu, Baosheng Guo and Qing Jiang**

Supporting Information

Inflammatory Fibroblast-Like Synoviocyte-Derived Exosomes Aggravate Osteoarthritis via Enhancing Macrophage Glycolysis

Bin Liu, Yansi Xian, Xiang Chen, Yong Shi, Jian Dong, Lin Yang, Xueying An, Tao Shen, Wenshu Wu, Yuze Ma, Yi He, Wang Gong, Rui Peng, Jiaquan Lin, Na Liu, Baosheng Guo, Qing Jiang**

Supplementary figures

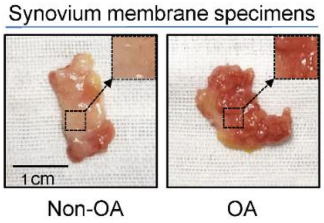

Figure S1. Macro morphology of the non-OA synovium and inflammatory OA synovium specimens. Scale bar, 1 cm.

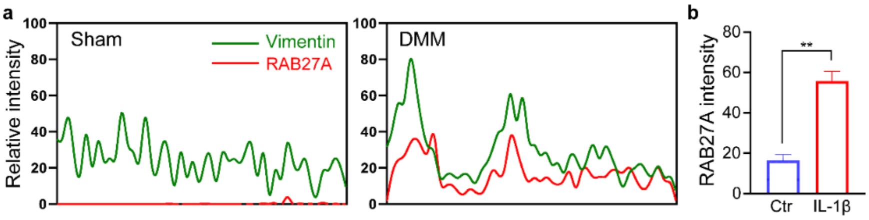

Figure S2. Relative quantification of RAB27A immunofluorescence. (a) Relative quantification of RAB27A immunofluorescence for synovium from sham-operated and DMM-operated knees. (b) Relative quantification of RAB27A immunocytochemical staining for mouse fibroblast-like synoviocytes with or without IL-1β stimulation. Data was presented as mean ± SD of three independent experiments. ** $P < 0.01$, versus the indicated groups, Student's *t*-test.

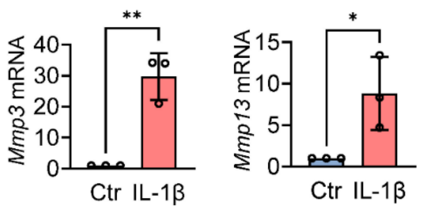

Figure S3. The mRNA level of *Mmp3* and *Mmp13* in intact and IL-1β stimulated mouse fibroblast-like synoviocytes (FLSs). n=3 for each group. ** $P < 0.01$, * $P < 0.05$, versus the indicated groups, Student's *t*-test.

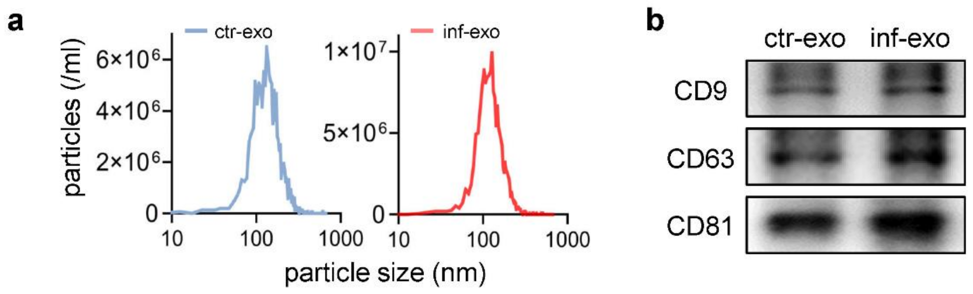

Figure S4. Characterization of ctr-exo and inf-exo. (a) Diameter distribution of ctr-exo and inf-exo determined using Nano Track Analysis (NTA). (b) Western blot confirmation of exosomal markers in ctr-exo and inf-exo.

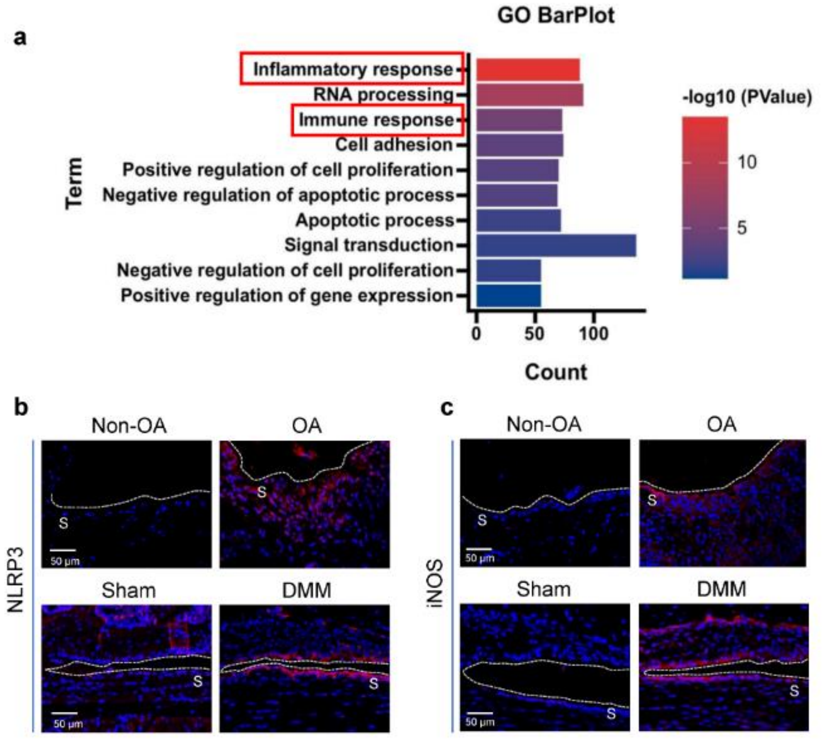

Figure S5. Inflammation is an important pathological feature of synovial lesions in OA. (a) Bar plot for top 10 enriched terms using GO enrichment analysis of differentially expressed genes between human OA and non-OA synovium. The raw transcriptome data was obtained from GSE89408 dataset. (b) and (c) Representative images of IF staining for NLRP3 and iNOS of OA or non-OA synovium specimens from patients and mice. Scale bar, 100 µm.

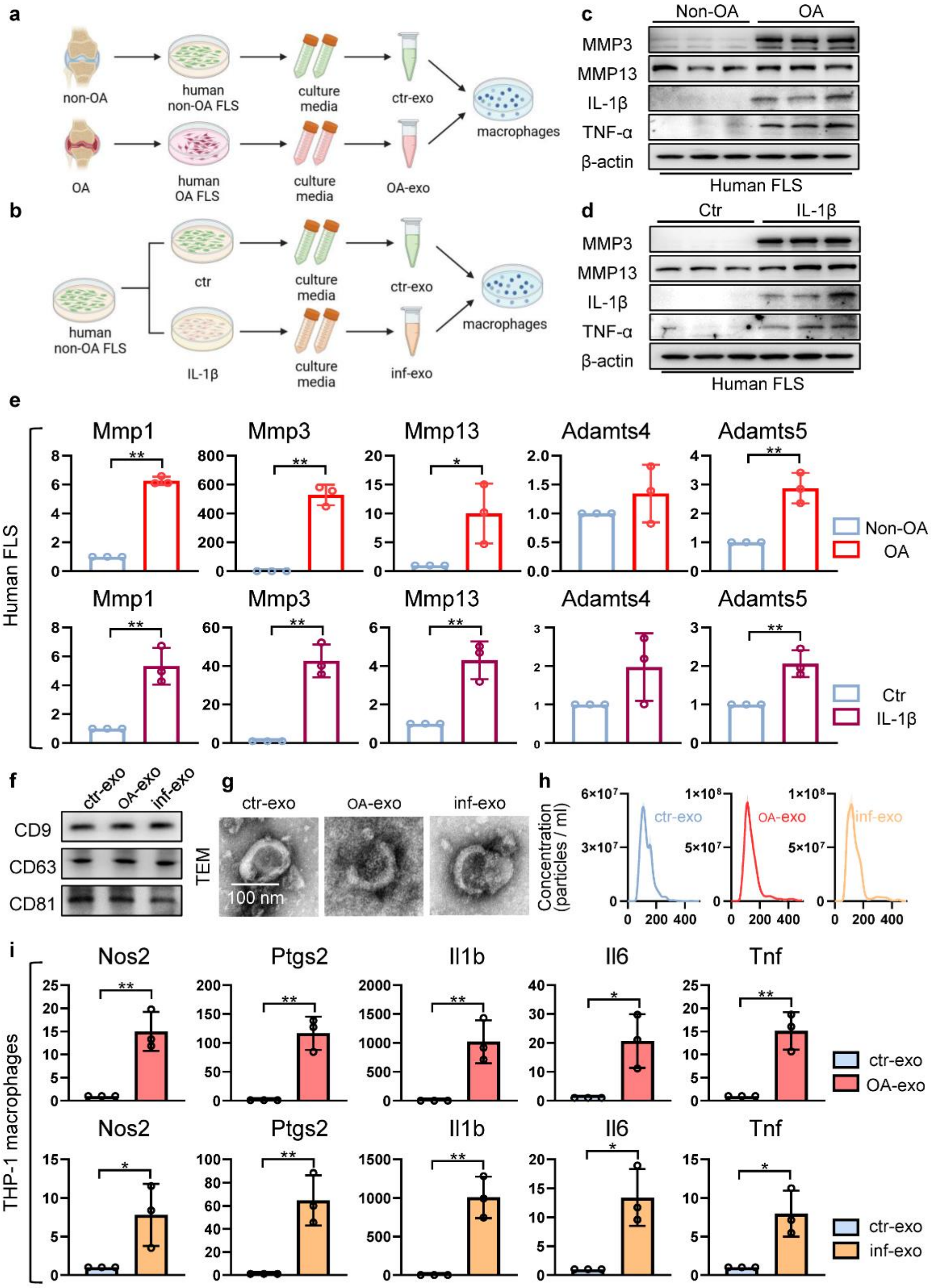

Figure S6. The effects of exosomes derived from human fibroblast-like synoviocytes (FLSs) on THP-1 cells. (a) and (b) Schemes for investigating the effects of exosomes derived from human FLSs on THP-1 cells. Primary human FLSs (passage 0) were isolated from human non-OA or OA synovium. The cell culture media of non-OA FLSs, OA FLSs, and IL-1β stimulated non-OA FLSs were collected for exosome isolation and the cells were collected for RNA and protein analysis. Exosomes from non-OA FLSs, OA FLSs, and IL-1β-stimulated FLSs were named ctr-exo, OA-exo, and inf-exo, respectively. (c), (d), and (e) Western blot and qPCR analysis of matrix degenerative enzymes and cytokines of FLSs. Western blot analysis (f), Transmission electron microscope (g), and Nano track analysis (h) for exosome characterization. (i) Expression of M1-related inflammatory genes in macrophages stimulated by different exosomes. n=3 for each group. ** $P < 0.01$, * $P < 0.05$, ns, not significant, versus the indicated groups, Student's *t*-test.

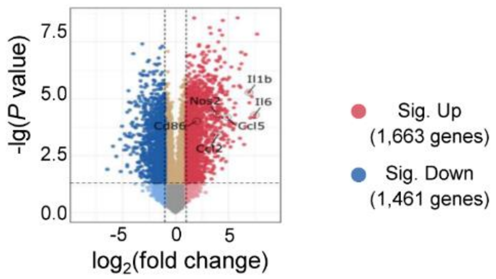

Figure S7. Volcano map of differentially expressed genes in macrophages after stimulation of the two exosomes. Volcano map showing 1,461 downregulated and 1,663 upregulated mRNAs in inf-exo treated macrophages compared to ctr-exo treated macrophages, fulfilling the criteria |log2 (fold change)| ≥ 1 and $P < 0.05$. The volcano map was generated using an online tool (www.omicstudio.cn).

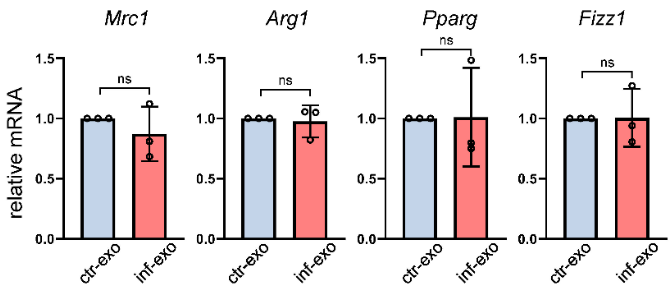

Figure S8. Expression of M2-related genes in macrophages stimulated by ctr-exo and inf-exo. n=3 for each group. ns, $P > 0.05$, versus the indicated groups, Student's t-test.

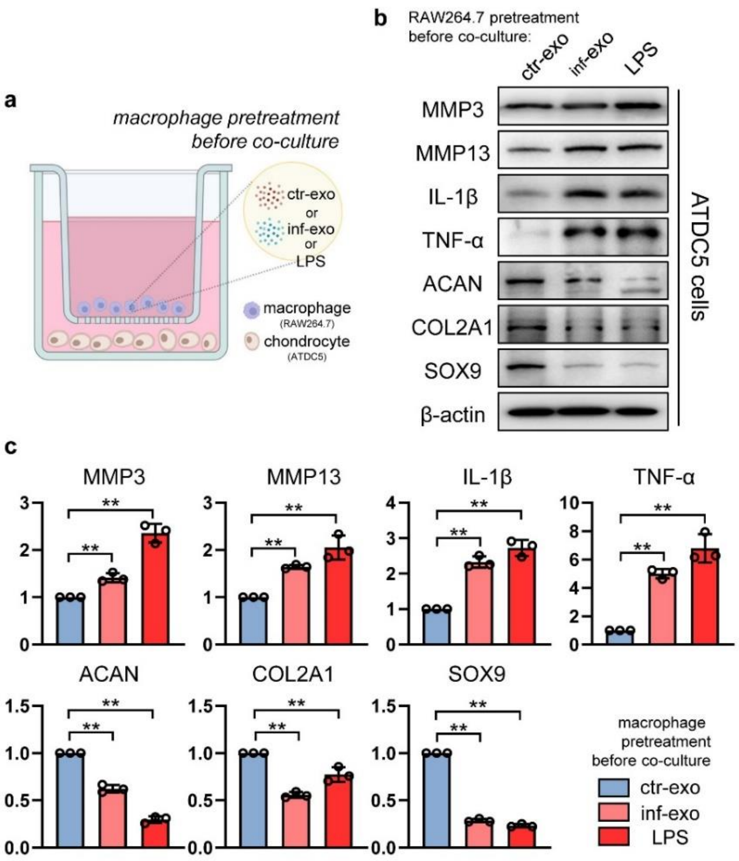

Figure S9. Similar effects of LPS-primed macrophages and inf-exo-primed macrophages on co-cultured chondrocytes. (a) Scheme for co-culture of macrophages and chondrocytes. The macrophages were stimulated with ctr-exo, inf-exo, and LPS (100 ng/mL) for 24 hours prior to co-culture and the chondrocytes were subjected to further western blot analysis. (b) and (c) Western blot analysis and corresponding quantification for chondrocytes after co-culture with macrophages. n=3 for each group, ** $P < 0.01$, versus the indicated groups, one-way ANOVA, Dunnett's multiple comparisons test.

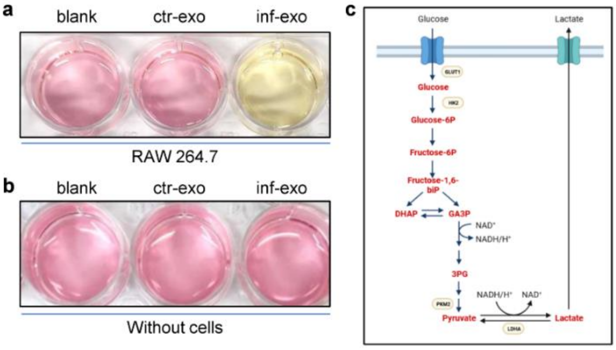

Figure S10. (a) and (b) Pictures of the cell culture media after exosome supplement for 48 hours, top panel with macrophages inoculated, bottom panel with medium only and no cells

for ruling out direct leaking of acidic cargo from exosomes. (c) Brief schematic diagram of glycolysis, containing major metabolic intermediates and flux-controlling enzymes.

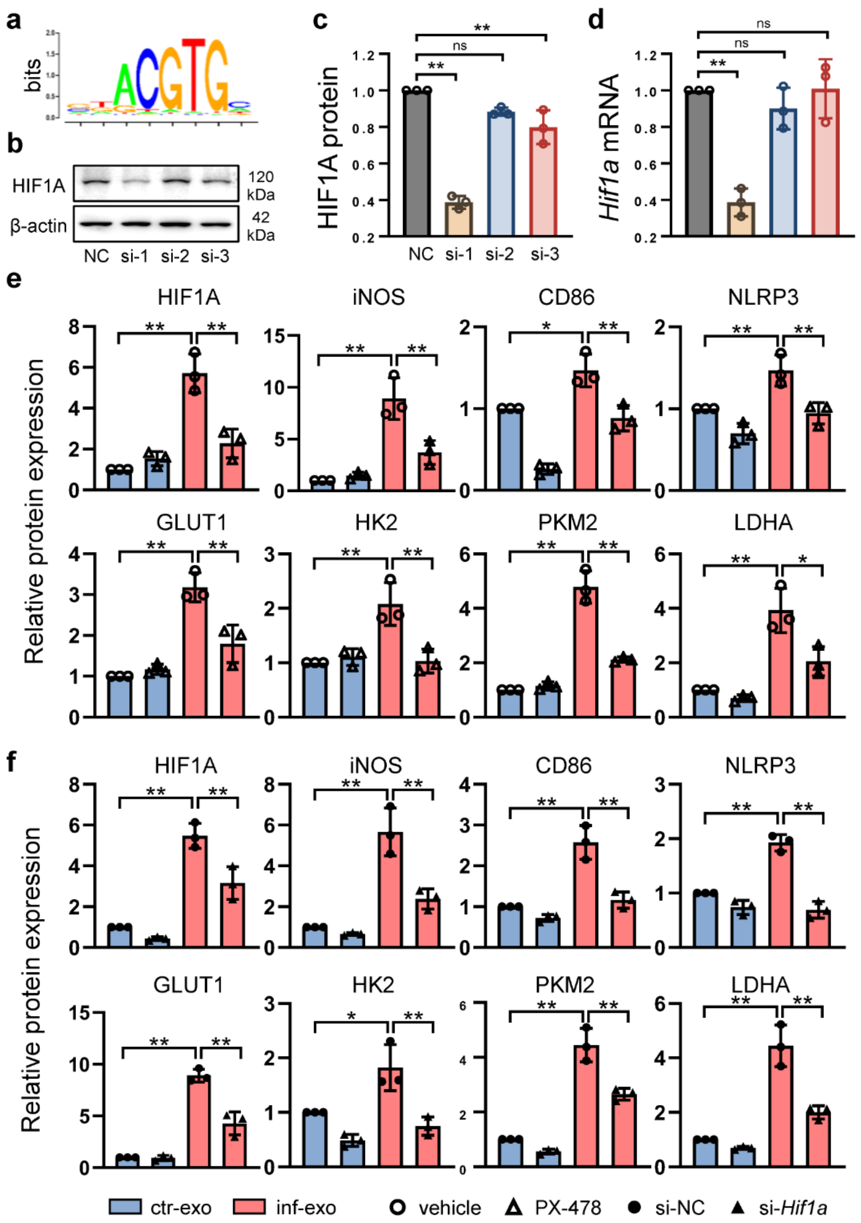

Figure S11. Involvement of HIF1A in inf-exo-triggered macrophage dysfunction. (a) Characteristic motif for HIF1A binding location (HRE) in the promoter regions. (b) and (c) Western blot analysis and quantification of HIF1A expression in macrophages after si-RNA or scramble sequence transfection. (d) Transcription level of *Hif1a* expression after si-RNA transfection. (e) and (f) Quantification of the M1-polarization-related protein and glycolysis enzymes in Fig. 6p, q. n=3 for each group. ** $P < 0.01$, * $P < 0.05$, ns, not significant, versus the indicated groups, Student's *t*-test.

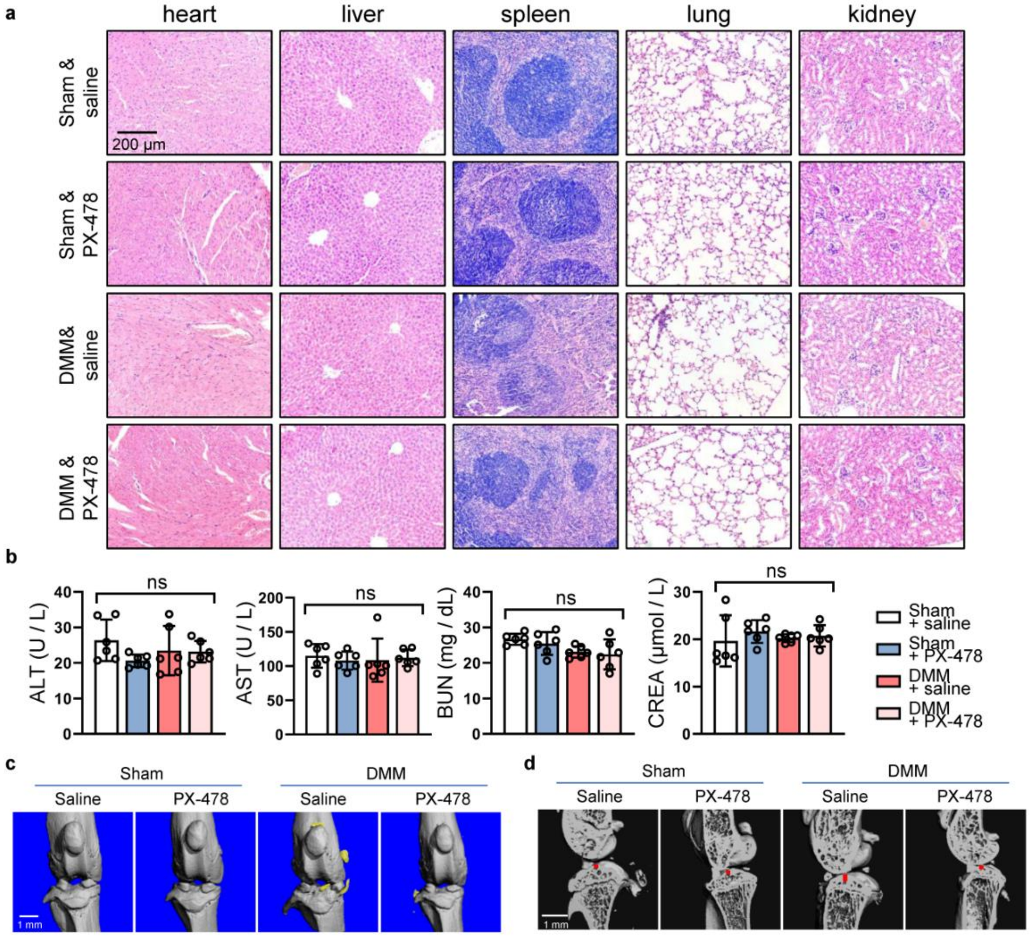

Figure S12. Safety of PX-478 treatment and μ-CT reconstruction of knees. (a) Representative H.E. staining images of important organs after 8-week PX-478 injection. (b) Histogram of serum alanine aminotransferase (ALT), aspartate aminotransferase (AST), blood urea nitrogen (BUN), and creatinine (CREA). n=6 for each group. ns, not significant, one-way ANOVA. (c) Three-dimensional reconstruction of μ-CT scanning of knee joints, with osteophytes labeled with yellow color. Scale bar, 1 mm. (d) The sagittal view of the knee joints, red line indicating the subchondral bone plate. Scale bar, 1 mm.

Table S1. Small interference RNA sequences for *Hif1a*

	Sense	Antisense
si-1	GCAGGAAUUGGAACAUUAUTT	AUAAUGUUCCAAUUCCUGCTG
si-2	CCAUGUGACCAUGAGGAAATT	UUUCCUCAUGGUCACAUGGAT
si-3	GCAGACCCAGUUACAGAAATT	UUUCUGUAACUGGGUCUGCTG

Table S2. Primers for chromosome immunoprecipitation polymerase chain reaction (ChIP-PCR)

	Forward	Reverse
Slc2a1	GCGGGAGACGCATAGTTACA	CAGCCCCGTTACTCACCTTG
Pkm2	ATTCAGCCTCCGCGTTCATC	CCCACCACCAAAAATCTGCG
Hk2	AGATTATGGCGTGTGCTGCT	CGTGTGTTCCGTGGCTAGAG
Ldha	AGGTGCAGGGTGTTCAGATT	CTCTTTACACGGGTCTCGCC

Table S3. Primers for quantitative real-time polymerase chain reaction (qRT-PCR) of mouse samples

Target	Forward	Reverse
Rab27a	TCGGATGGAGATTACGATTACCT	TTTTCCCTGAAATCAATGCCCA
Il1b	GAAATGCCACCTTTTGACAGTG	TGGATGCTCTCATCAGGACAG
Il6	TAGTCCTTCCTACCCCAATTTCC	TTGGTCCTTAGCCACTCCTTC
Tnf	CCCTCACACTCAGATCATCTTCT	GCTACGACGTGGGCTACAG
Nos2	GTTCTCAGCCCAACAATACAAGA	GTGGACGGGTCGATGTCAC
Cd86	GGTGGCCTTTTTGACACTCTC	TGAGGTAGAGGTAGGAGGATCTT
Slc2a1	CAGTTCGGCTATAACACTGGTG	GCCCCCGACAGAGAAGATG
Pkm2	GCCGCCTGGACATTGACTC	CCATGAGAGAAATTCAGCCGAG
Hk2	TGATCGCCTGCTTATTCACGG	AACCGCCTAGAAATCTCCAGA
Ldha	TGTCTCCAGCAAAGACTACTGT	GACTGTACTTGACAATGTTGGGA
Ccnd1	GCGTACCCTGACACCAATCTC	CTCCTCTTCGCACTTCTGCTC
Ptgs2	TTCAACACACTCTATCACTGGC	AGAAGCGTTTGCGGTACTCAT
Mmp3	ACATGGAGACTTTGTCCCTTTTG	TTGGCTGAGTGGTAGAGTCCC
Mmp13	CTTCTTCTTGTTGAGCTGGACTC	CTGTGGAGGTCACTGTAGACT
Bax	TGAAGACAGGGGCCTTTTTG	AATTCGCCGGAGACACTCG
Bcl2	GTCGCTACCGTCGTGACTTC	CAGACATGCACCTACCCAGC
Mrc1	CTCTGTTCAGCTATTGGACGC	CGGAATTTCTGGGATTCAGCTTC
Arg1	CTCCAAGCCAAAGTCCTTAGAG	AGGAGCTGTCATTAGGGACATC
Pparg	GGAAGACCACTCGCATTCCTT	GTAATCAGCAACCATTGGGTCA
Fizz1	AAGCCTACACTGTGTTTCCTTTT	GCTTCCTTGATCCTTTGATCCAC
Actb	GGCTGTATTCCCCTCCATCG	CCAGTTGGTAACAATGCCATGT

Table S4. Primers for quantitative real-time polymerase chain reaction (qRT-PCR) of human samples

Target	Forward	Reverse
Il1b	ATGATGGCTTATTACAGTGGCAA	GTCGGAGATTCGTAGCTGGA
Il6	ACTCACCTCTTCAGAACGAATTG	CCATCTTTGGAAGGTTCAGGTTG
Tnf	CCTCTCTCTAATCAGCCCTCTG	GAGGACCTGGGAGTAGATGAG
Nos2	TTCAGTATCACAACCTCAGCAAG	TGGACCTGCAAGTTAAAATCCC
Ptgs2	CTGGCGCTCAGCCATACAG	CGCACTTATACTGGTCAAATCCC
Mmp1	GGGGCTTTGATGTACCCTAGC	TGTCACACGCTTTTGGGGTTT
Mmp3	CGGTTCCGCCTGTCTCAAG	CGCCAAAAGTGCCTGTCTT
Mmp13	ACTGAGAGGCTCCGAGAAATG	GAACCCCGCATCTTGGCTT
Adamts4	GAGGAGGAGATCGTGTTTCCA	CCAGCTCTAGTAGCAGCGTC
Adamts5	GGCCTCCATCGCCAATAGG	GGATAGCTGCATCGTAGTGCT
Actb	CATGTACGTTGCTATCCAGGC	CTCCTTAATGTCACGCACGAT
